# Supplementary material for: Intrinsically fluorescent polyureas toward conformation-assisted metamorphosis, discoloration and intracellular drug delivery
Source: Nat Commun. 2022 Aug 5;13:4551. doi: 10.1038/s41467-022-32053-1 (PMC9355952; doi:10.1038/s41467-022-32053-1)
Supplement: Supplementary file 1 — Supporting Information [file 41467_2022_32053_MOESM1_ESM.pdf]

Supporting Information for

Intrinsically Fluorescent Polyureas toward Conformation-Assisted Metamorphosis, Discoloration and Intracellular Drug Delivery

Yeqiang Zhou[1,2], Fan Fan[1,2], Jinling Zhao[1], Zhaoding Wang[1], Rui Wang[1], Yi Zheng[1], Hang Liu[1], Chuan Peng[1], Jianshu Li[1], Hong Tan[1], Qiang Fu[1], Mingming Ding[1,*]

[1]*College of Polymer Science and Engineering, State Key Laboratory of Polymer Materials Engineering, Sichuan University, Chengdu 610065, China.* [2]*These authors contributed equally: Yeqiang Zhou, Fan Fan. * Corresponding author, email: dmmshx@scu.edu.cn*

Content

Materials..........2

Supplementary Methods..........2

Supplementary Figures..........7

Supplementary Tables..........48

Supplementary References..........48

Materials

L-Cystine dimethyl ester dihydrochloride (L-Cys OMe·2HCl, 98%) and D-cystine dimethyl ester dihydrochloride (D-Cys OMe·2HCl, 98%) were obtained from Shanghai Hanhong Chemical Technology Co., Ltd. (Shanghai, China). L-Lysine ethyl ester diisocyanate (LDI, 98%) was purchased from Nantong Dahong Chemical Co. Ltd. (Nantong, China). mPEG (Mn = 5000) was obtained from Adams Reagent Co., Ltd. (Shanghai, China). L-lysine ethyl ester dihydrochloride (L-Lys OEt·2HCl; 98%) was obtained from Gill Biochemical Co., Ltd. (Shanghai, China). Stannous octoate was purchased from TEDA Letai Chemical Co., Ltd. (Tianjin, China). Rhodamine 6G (R6G) was purchased from TCI (Shanghai) Chemical Industry Development Co., Ltd. (Shanghai, China). Methanol, trifluoroethanol (TFE), trypsin and sodium lauryl sulphate (SDS, 85%), trichloromethane ($CHCl_3$), *N*,*N*-dimethyl formamide (DMF) and *N*,*N*-dimethylacetamide (DMAC) were obtained from Chengdu Kelong Chemical Co., Ltd. (Chengdu, China). Doxorubicin hydrochloride (DOX·HCl) was obtained from Meilun Biological Products Co., Ltd. (Dalian, China). Glutathione (GSH, 98%) was purchased from Biofrox (Guangzhou, China). Perdeuterated dimethylsulfoxide (DMSO-d_6) and thioflavin T (ThT) were obtained from Sigma-Aldrich (Shanghai, China). The MCF-7 cell lines were purchased from the cell culture center of the Institute of Basic Medical Sciences, Chinese Academy of Medical Sciences (Beijing, China). Trifluoroacetate (TFA) was purchased from Titan scientific Co., Ltd (Shanghai, China). DMF and DMAC were stirred over CaH_2 (5% w/w), then distilled at 20 mm Hg and stored over 4 A molecular sieves. Unless stated elsewhere, other reagents were obtained from commercial suppliers and used without further purification.

Supplementary Methods

Proton nuclear magnetic resonance spectroscopy (^{1}H NMR, 400 MHz) and carbon-13 nuclear magnetic resonance spectroscopy (^{13}C NMR, 400 MHz) were recorded on a Bruker Avance III HD spectrometer using tetramethylsilane (TMS) as an internal standard and DMSO-d_6 as solvents at room temperature.

The two-dimensional nuclear Overhauser effect spectroscopy (2D NOESY) spectra were measured using an AVANCE III HD spectrometer (400 MHz, JEOL) with a sweep width of 4000 Hz into 1024 data points. The relaxation delay was 2 s and the mixing time was 0.3 s. The number of scans was 4.

Fourier transform infrared (FTIR) spectroscopy was recorded on a Nicolet iS10 spectrometer (Thermo Electron Corporation, U.S.A) from 4000 to 600 cm^{-1}. The sample solutions were cast on KBr plates and dried under vacuum before measurement. FTIR absorption peaks of PUs in the ranges of 3600-3100 cm^{-1}, 1770-1690 cm^{-1}, 1690-1600 cm^{-1} were was treated by using a Gaussian function curve-fitting analysis.[1]

Gel permeation chromatography (GPC) was carried out on an HLC-8320 (TOSOH Corporation, Japan) at 40 °C using DMF/LiBr (2 g L^{-1}) as an eluent and polymethyl methacrylate (PMMA) as a standard. The sample concentration was 2 mg mL^{-1} and the flow rate was 1.0 mL min^{-1}.

For cryo-scanning electron microscopy (cryo-SEM), the PU assemblies were vitrified with liquid nitrogen and maintained at -140 ℃. First, a small amount of sample was placed into a slot on a stub with rivets and cryo-vitrified with liquid nitrogen. Thereafter, the vitrified PUs were transferred onto a cryo-stage (at -140 ℃) in the preparation chamber (PP3010T cryo-SEM

Preparation System, Quorum Technologies, UK). After 15–30 min the PUs were sputtered with platinum using argon gas to prevent charging during electron beam targeting. Lastly, the samples were transferred on to the SEM stage (JEOL JSM 7100F SEM, JEOL Ltd, Tokyo, Japan) at -140 ℃ for observation.

Field emission transmission electron microscope (TEM) was conducted on a Tecnai G2 F20 S-TWIN electron microscope. The PU assemblies (40 μL, 0.5 mg mL^{-1}) were kept in an aqueous solution for 24 h and dropped on copper meshes. After 5 min, unnecessary liquid was removed and phosphotungstic acid solution (5 μL) was dropped on this copper mesh to stain the samples for 5 min. Finally, the copper meshes were dried at room temperature for observation.

Fluorescence measurement was conducted on an F-4600 FL spectrophotometer (Hitachi, Ltd., Japan). For pyrene fluorescence, the excitation spectra were collected from 200 to 360 nm at an emission wavelength (λ_{em}) of 372 nm, and the fluorescence emission spectra was recorded at an excitation wavelength (λ_{ex}) of 334 nm. For R6G fluorescence, the emission spectra were collected from 530 to 700 nm at a λ_{ex} of 526 nm. For PU fluorescence, the emission spectra were collected from 380 to 700 nm at a λ_{ex} of 365 nm. For DOX fluorescence, the emission spectra were collected from 500 to 800 nm at a λ_{ex} of 480 nm. For quinacrine dihydrochloride (QD) fluorescence, the emission spectra were collected from 440 to 700 nm at a λ_{ex} of 430 nm.

UV–vis absorbance spectra were obtained using a UV2600 spectrophotometer (Techcomp, Ltd., China) and a quartz cuvette having an optical path length of 1.00 cm. Depending on the samples, the same solvent as sample solution was used for background subtraction.

Dynamic light scattering (DLS) was performed on a Malvern nano-zeta sizer instrument (Malvern Instruments Ltd., UK) at room temperature at an angle of 90 °. The sizes are reported as the hydrodynamic diameter (D_H) and each measurement was repeated thrice. 0.5 mg mL^{-1} PU assemblies were filtered using a hydrophilic membrane (pore size 0.450 μm) before the experiment was performed. The relevant data were presented as mean ± standard deviation (SD) based on triplicate independent experiments.

All the calculations were based on an all-atom molecular dynamics (AAMD) simulation performed using a Gromacs package.[2] System configurations were visualized using VMD software, The general OPLS force field was used to model the P_4 and P_2 molecules. Water molecules were modeled using the tip4p potential. In this paper, we performed AAMD simulations on the selected model system consisting of 3 P_4 and 3 P_2 in a water box sized 136.8 × 136.8 × 136.8 nm^3 at neutral states.

The structures of PUs were characterized by ^{1}H NMR and FTIR. As shown in Supplementary Fig 3, the characteristic peaks of methylene protons and methyl protons in the side chains of LDI residues could be found at 4.08 (COO-C$\boldsymbol{H}_2$-CH_3) and 1.01 ppm (COO-CH_2-C$\boldsymbol{H}_3$), respectively. The signal of methyl in Cys OMe (COO-C$\boldsymbol{H}_3$) was observed at 3.66 ppm. The two peaks at 3.24 (CH-C$\boldsymbol{H}_2$-S-S-CH_2-CH) and 3.06 ppm (CH-CH_2-S-S-C$\boldsymbol{H}_2$-CH) are ascribed to the methylene protons next to disulfide linkage in Cys OMe residues. The chemical shifts at 2.96 (NH-C$\boldsymbol{H}_2$-CH_2-CH_2-CH_2), 1.25 (NH-CH_2-C$\boldsymbol{H}_2$-CH_2-CH_2) at 1.54 (NH-CH_2-CH_2-C$\boldsymbol{H}_2$-CH_2) and 1.94 (NH-CH_2-CH_2-CH_2-C$\boldsymbol{H}_2$) ppm are assigned to the methylene groups of LDI residues in the polymeric backbone. The proton of methine near the amide bond (NH-C***H***-CO) could be found at 4.55 ppm. In addition, the block numbers of PUs were 21~81 as calculated from the integral area of the ^{1}H NMR peaks at 3.50 (mPEG), 4.08 (LDI) and 3.66 (Cys OMe) ppm. The FTIR spectra of PUs were depicted in Supplementary Fig. 7 The presence of urea C=O stretching vibration band

(1600-1700 cm^{-1}) and the disappearance of isocyanate signal (2270 cm^{-1}) confirm the successful synthesis of PUs. The strong adsorption peak around 3390 cm^{-1} was assigned to NH stretching vibration. The absorption band at 1560 cm^{-1} was attributed to NH plane banding vibration. The band from 1718 to 1744 cm^{-1} corresponds to the ester carbonyl stretching vibration.[3, 4]

Fluorescence Quantum yield (FQY) measurement. The FQYs of PUs were measured in reference to quinine sulfate in 0.1 M H_2SO_4 (literature quantum yield 54% at 350 nm excitation). The same excitation wavelength and slit band widths were applied for the two samples. The formula used for FQY measurements was as follows.

$$\mathrm{FQY} = (I / I_\mathrm{R}) \times (A_\mathrm{R} / A) \times (\eta / \eta_\mathrm{R})^2 \times \mathrm{FQY}_\mathrm{R} \qquad (1)$$

where FQY is the quantum yield of the sample, I is the integral area under the fluorescence spectrum, η is the refractive index of the solvent used and A is the absorbance at the excitation wavelength. The subscript R represents the reference. to minimize reabsorption effects, absorbencies were kept under 0.05 at the excitation wavelength of 350 nm. the FQYs of PUs and DL-PUs were measured and presented in Supplementary Fig. 31. The FQY of PUs is up to 29%, which is much higher than those of the reported polysiloxanes, poly(amino esters) and poly(urea-urethane)s.[5-8]

R6G encapsulation study. A solution of R6G in deionized water (120 μL, 0.05 mg mL^{-1}) was added dropwise into 2 mL of assembled solutions prepared from P_1, P_2, P_3 and P_4 (0.2 mg mL^{-1}) with constant stirring for 2 h. After that, the solutions were ultrasonated for 2 h, transferred to a dialysis bag (MWCO 3500) and dialyzed against water for 24 h, changing the water every 3 h. The UV-Vis spectra were taken and the concentration of free R6G solution in water was adjusted so that the UV-Vis absorption matched the intensity of R6G encapsulated in assemblies (Fig.1d). The fluorescence emission spectra of R6G in water and assembled solutions were obtained at λ_{ex} of 526 nm on an F-4600 FL spectrophotometer (Hitachi, Ltd., Japan).

Fluorescence resonance energy transfer (FRET) study. The assembly of PUs was prepared via a dialysis method, Meanwhile, Samples for the FRET studies were prepared by adding appropriate concentration of the quinacrine dihydrochloride (QD) in deionized water (0.2 mL) to 3 mL PU assemblies with constant stirring for 1 h, and then ultrasonated for 2 h. the aqueous solution was transferred to a dialysis bag (MWCO 3500) and dialyzed against water for 24 h, changing the water every 3 h. Then, the emission spectra were recorded on an F-4600 FL spectrophotometer (Hitachi, Ltd., Japan).

The FRET efficiency was calculated from the donor fluorescence quenching profiles by using the equation (2).[9, 10]

$$\text{FRET efficiency} = I_\mathrm{QD} / (I_\mathrm{PU} + I_\mathrm{QD}) \qquad (2)$$

where I_{QD} and I_{PU} are the fluorescence intensities at 493 and 420 nm, respectively.

Small Angle X-ray Scattering (SAXS). SAXS measurements were conducted on a Xeuss 2.0 system (Xenocs SA, Grenoble, France) with a microfocused Cu Kα source and a Rayonix MX225-HE CCD X-ray detector. The beamline was operated at 15 keV corresponding to a wavelength of 0.83 Å. For measurement, 100 μL of PU dispersion in water was transferred into a quartz glass capillary with a diameter of 1.5 mm. The sample-to-detector distance was set as 3489.2 mm to collect data from q = 0.0042 ~ 0.114 $Å^{-1}$ range. The length of scattering vector q was defined as $q = 4\pi\sin\theta/\lambda$, where θ is half of the angle between incident and scattered X-rays, λ is the wavelength of the X-ray. scattering from water was recorded in the same way for background subtraction. The single spectra were averaged and subtracted for background using the

FOXTROT software. The SAXS curve was fitted with a SasView software using a hollow cylinder model and vesicle model.[11-13]

Pyrene fluorescence probe study. A solution of pyrene in acetone (20 μL, 5.0 $\times 10^{-6}$ mol L^{-1}) was transferred into a series of vials and acetone was evaporated under argon flow. Then 2 mL of PU assemblies with different concentrations were added into the vials and ultrasonated for 2 h. The steady-state fluorescence emission spectra were recorded on an F-4600 FL spectrophotometer at λ_{ex} of 365 nm, with bandwidths of 5 nm for excitation and 5 nm for emission. Hydrophobic pyrene is a sensitive and effective fluorescent probe.[14, 15] When the hydrophobic microdomain forms in water during self-assembly, pyrene will transfer from a hydrophilic environment to a hydrophobic environment, leading to changes in the intensity and position of the peaks in fluorescence spectra.[16, 17] As shown in Supplementary Fig. 17, with an increase of PU concentrations in water, the peak intensity ratios (I_{374}/I_{384}) in the emission spectra almost unchanged, while the peaks at 334 nm in the excitation spectra did not moved, indicating that pyrene molecules cannot enter the hydrophobic environment due to the formation of tight hydrogen bond in the vesicular membrane.

TFA treatment. PU assemblies were treated with different concentration of TFA, a strong breaker of hydrogen bond.[18] The morphologies and size of PU assemblies after treatment were monitored by DLS, TEM and CLSM. For CLSM, hydrophilic DOX·HCl and hydrophobic FITC were loaded into the assemblies. In brief, a solution of DOX·HCl in water (150 μL, 0.05 mg mL^{-1}) was added dropwise into 1 mL of assembled solutions prepared from P_1, P_2, P_3, and P_4 (2 mg mL^{-1}). Afterward, the solution was stirred for 0.5 h. After extensive dialysis (MWCO 3500) for 24 h to remove free DOX·HCl, 150 μL of FITC in acetone (0.05 mg mL^{-1}) was added dropwise into the solutions. Free dyes and acetone were then removed by dialysis (MWCO 3500) against water, centrifugalized at 3000 r min^{-1} for 10 min and passed through a 0.45 mm pore-sized syringe filter (Millipore, Carrigtwohill, Co. Cork, Ireland). The resulting fluorescent-loaded assemblies were imaged by confocal laser scanning microscope (CLSM, Nikon A1RMP, Japan).

To investigate the reversibility of morphological transition, the sizes and size distributions of PU assemblies treated with TFA were monitored with a Zetasizer Nano ZS instrument (Malvern Instruments Ltd., UK) at room temperature at an angle of 90 °, and the fluorescence intensity of the samples were monitored with an F-4600 FL spectrophotometer (Hitachi, Ltd., Japan) at λ_{ex} of 365 nm. After removal of TFA by extensive dialysis (MWCO 3500), the solution was adjusted to a constant volume and determined again with DLS and fluorescence spectrophotometer. The removal and addition of TFA were repeated many times for the measurements. In addition, to verify the integrity of PU structures during TFA treatments, the polymers were incubated in TFA for one week and analyzed with ^{1}H NMR. As found in Supplementary Fig. 18, the characteristic peaks and their integral areas were kept nearly unchanged after incubation with TFA, suggesting that the TFA treatment did not disrupt the molecular integrity of PUs.

Stability of PU assemblies. To investigate the stability of PU assemblies under physiological conditions, taking P_3 as an example and a diblock copolymer of mPEG and poly(ε-caprolactone) (mPEG-PCL) as a control, the polymeric assemblies were diluted with deionized water, or treated with methanol or SDS (0.02 M) with shaking. Then the assemblies were examined on a Zetasizer Nano ZS instrument (Malvern Instruments Ltd., UK) at a scattering angle of 90 °.

MTT assay. To evaluate the cytotoxicity of drug-free and drug-loaded PU assemblies, MCF-7 cells were seeded in 96-well plates at a density of 5×10^3 cells per well and cultured overnight.

Then the samples with different concentrations were added separately into the plates and incubated for 24 and 72 h. Afterward, 20 μL of MTT solution (5mg mL^{-1}) was added into each well for another 2 h of incubation. Finally, the solution was replaced by 200 μL of DMSO. After shaking the plates for 10 min to dissolve the formazan crystals, the absorption intensity at 490 nm was recorded on a microplate reader (DNM-9602, Nanjing Perlove Medical Equipment Co., Ltd., China).

Construction of tumor model. Five to six-week-old female BALB/c nude mice were purchased from Vital River Company in Beijing. All experimental procedures were in accordance with the guidelines for laboratory animals established by the Laboratory Animal Center of Sichuan University. MCF-7 cell line was cultured in Dulbecco's Minimal Eagle Medium (DMEM) medium with 10% fetal bovine serum. 100 μL of cell suspensions (2.0×10^7 cells mL^{-1}) were injected into the upper right flank of the mice. The body weights and tumor sizes were measured every 3 d. The tumor volume was calculated using the equation $V = ab^2/2$, where "a" and "b" represent the length and width of tumors, respectively.

In vivo and ex vivo imaging study. To investigate the targeting property and biodistribution of PU assemblies in vivo, MCF-7 tumor-bearing nude mice were randomly divided into three groups. When the tumors had grown to around 100 mm^3, the mice were intravenously injected with DOX@PU assemblies *via* the tail vein, and tracked by an IVIS imaging system (Caliper Life Sciences, USA) at different time points. The excitation filter is 490 nm, and the emission filter are 600 and 700 nm. The animals were sacrificed at 24 h post-administration, and tumor tissues and major organs including heart, liver, spleen, lung, and kidney were collected for ex vivo fluorescence examination using the same imaging system.

Supplementary Figures

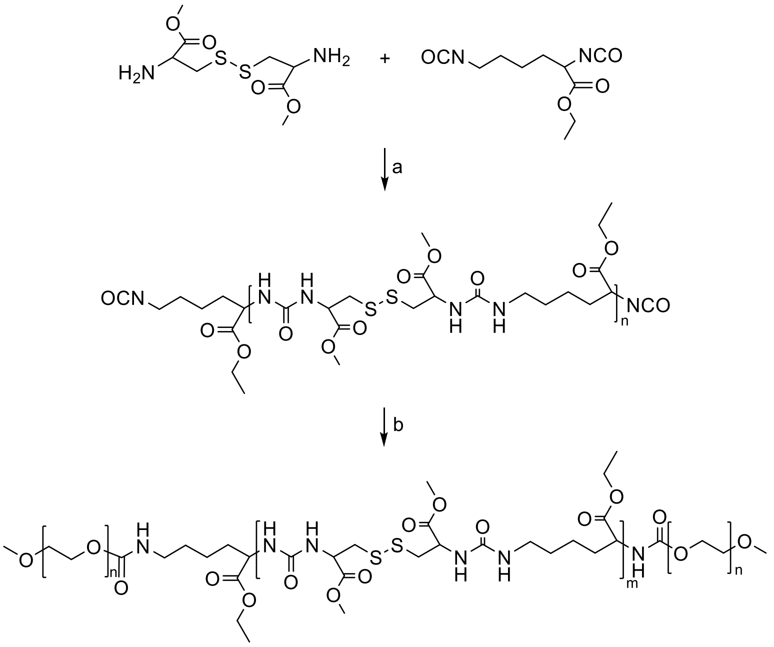

Supplementary Fig. 1 Synthesis of PUs and DL-PUs (P_1, P_2, P_3, P_4, DL-P_1, DL-P_4) using L-Cys OMe·2HCl, D-Cys OMe·2HCl, L-Lys OEt·2HCl and LDI as monomers and mPEG5000 as an end-caping agent. Reagents and conditions: (**a**) TEA, DMAC, 60 ℃, 2 h; (**b**) mPEG5000, stannous octanoate, 80 ℃, 3 d, dialysis, lyophilization (80-90% yield).

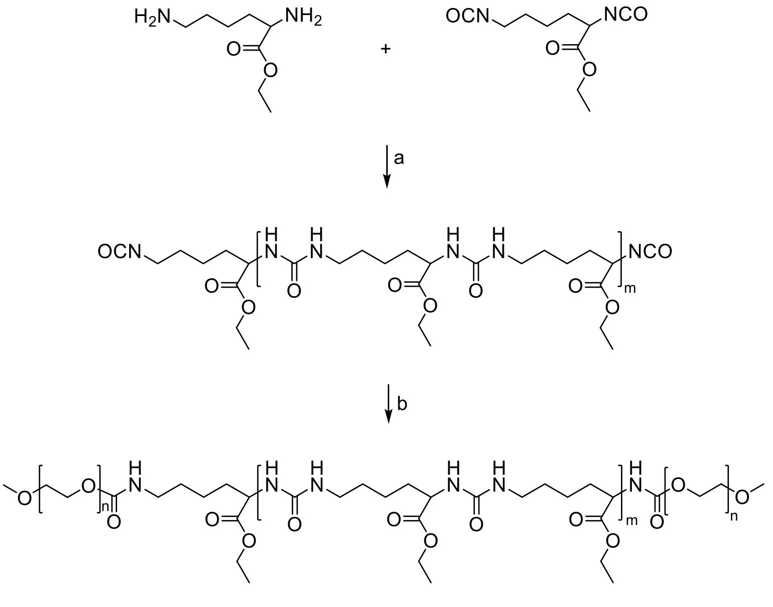

Supplementary Fig. 2 Synthesis of P_5 using L-Lys OEt·2HCl and LDI as monomers and mPEG5000 as an end-caping agent. Reagents and conditions: (**a**) TEA, DMAC, 60 ℃, 2 h; (**b**) mPEG5000, stannous octanoate, 80 ℃, 3 d, dialysis, lyophilization (90% yield).

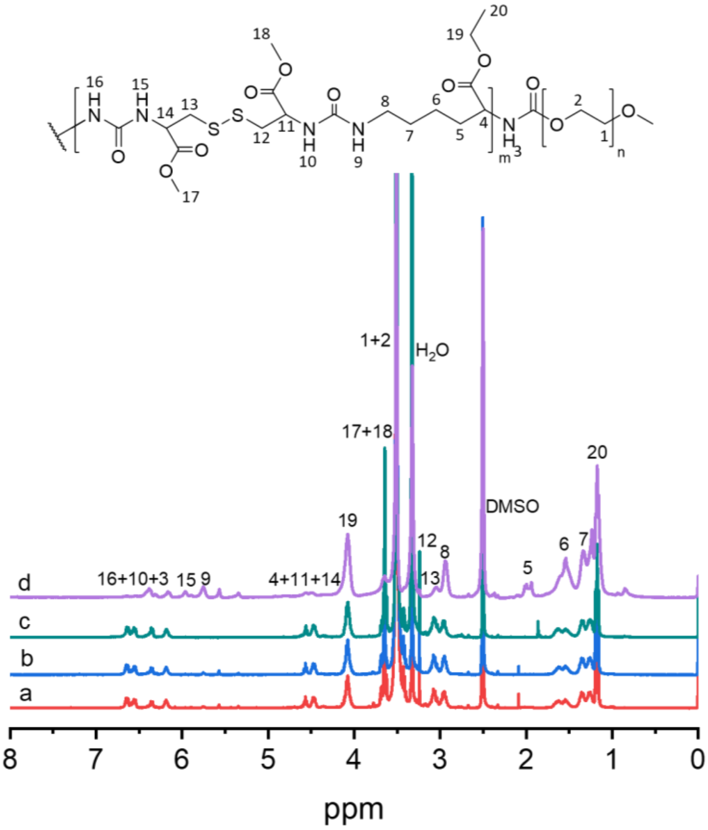

Supplementary Fig. 3 ^{1}H NMR spectra (400 MHz) of PUs in DMSO-*d*$_6$: (**a**) P_1; (**b**) P_2; (**c**) P_3; (**d**) P_4.

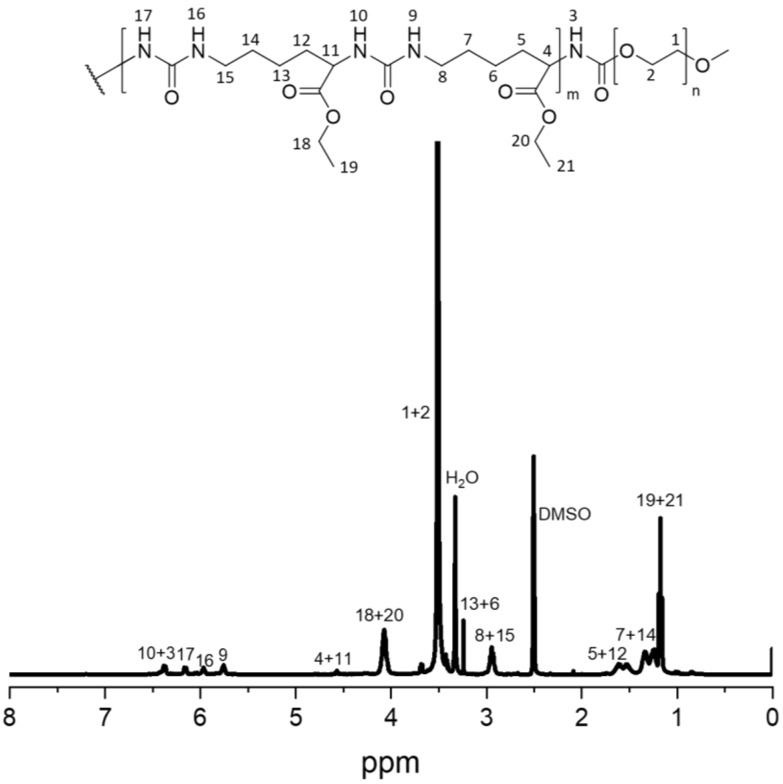

Supplementary Fig. 4 ^{1}H NMR (400 MHz) spectrum of P_5 in DMSO-d_6.

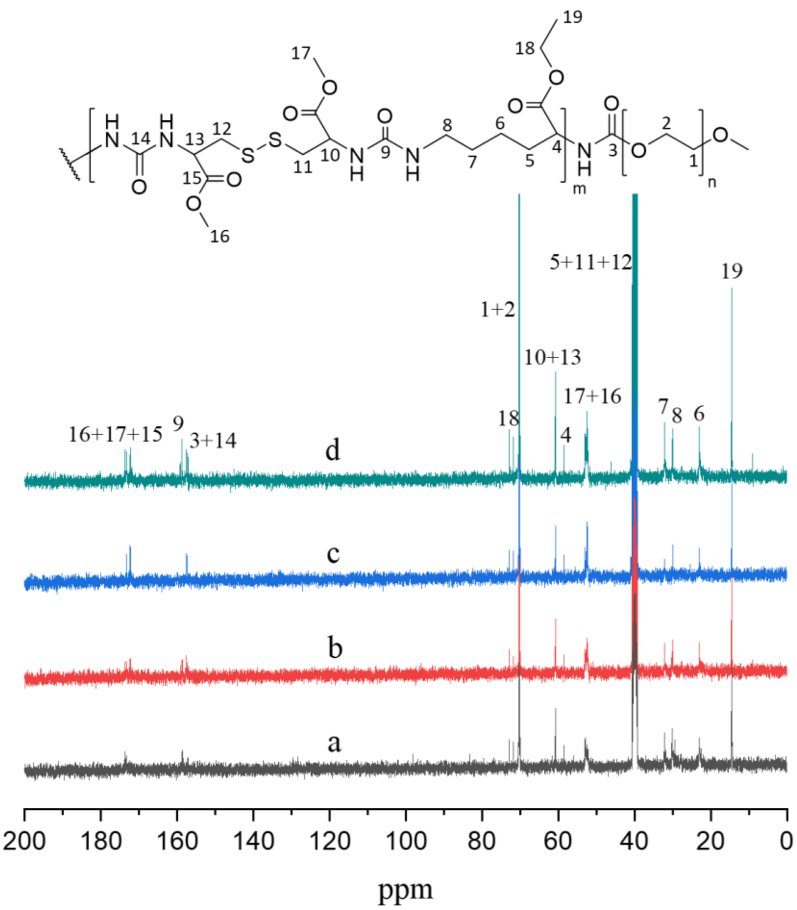

Supplementary Fig. 5 ^{13}C NMR (400 MHz) spectra of PUs in DMSO-d_6: (**a**) P_1; (**b**) P_2; (**c**) P_3; (**d**) P_4.

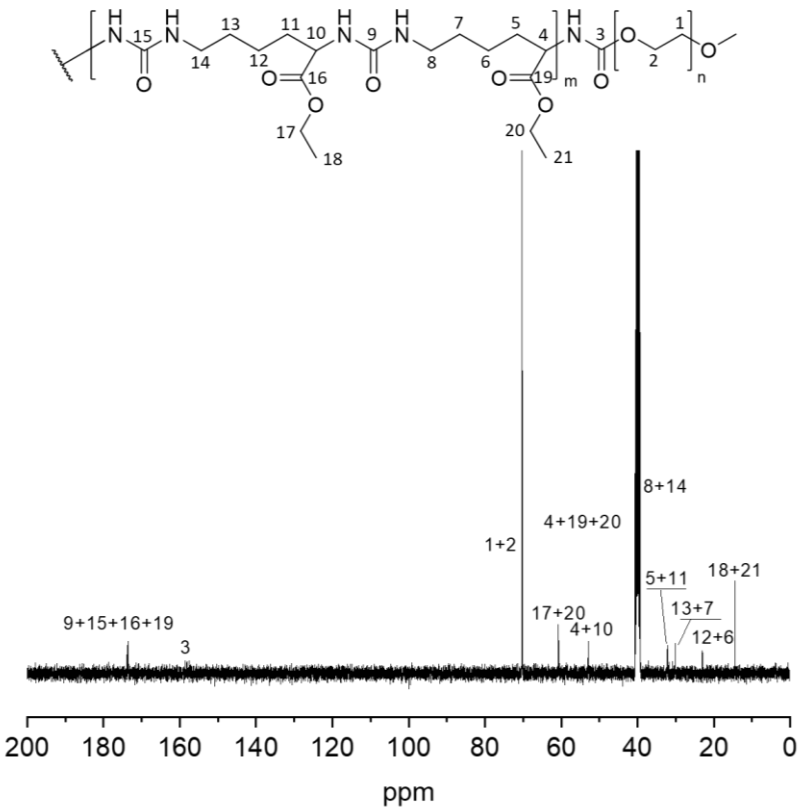

Supplementary Fig. 6 ^{13}C NMR (400 MHz) spectrum of P_5 in DMSO-d_6.

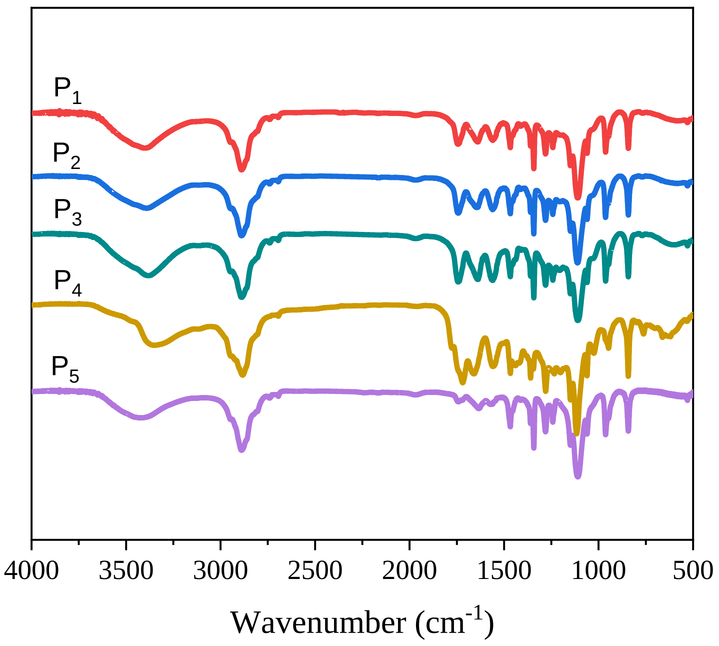

Supplementary Fig. 7 FTIR spectra of PUs (500-4000 cm^{-1}).

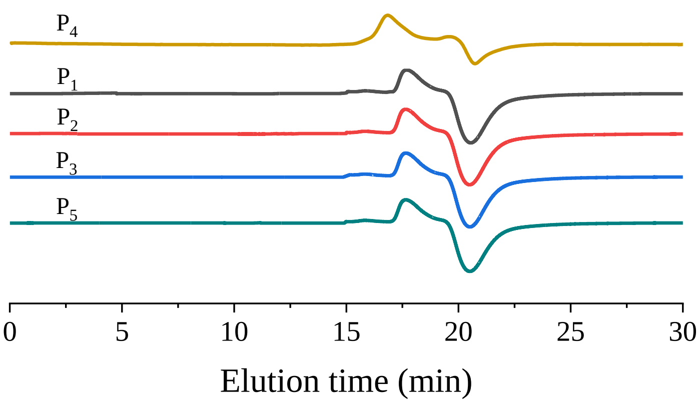

Supplementary Fig. 8 GPC chromatograms of PUs in DMF/LiBr (2 g L^{-1}).

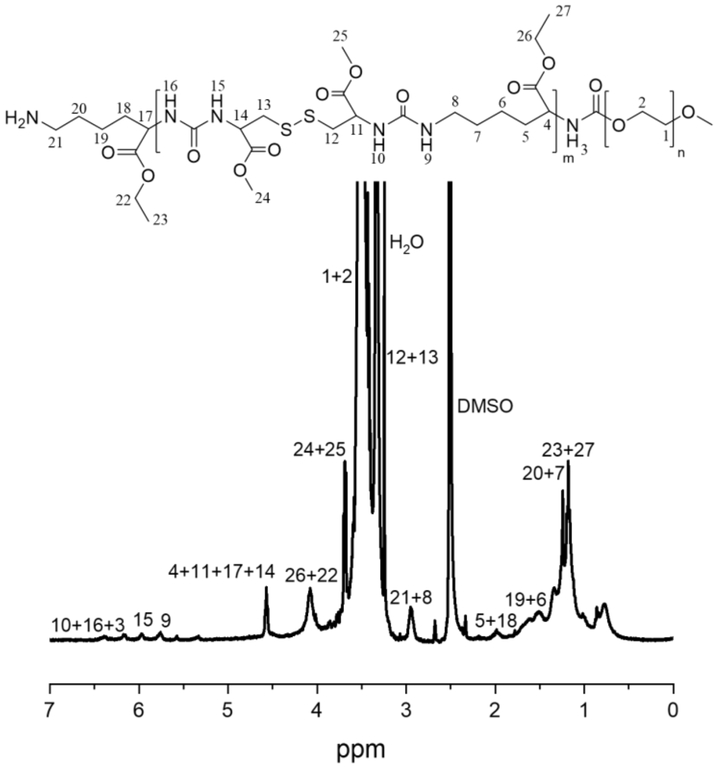

Supplementary Fig. 9 ^{1}H NMR spectrum (400 MHz) of DTPU in DMSO-d_6.

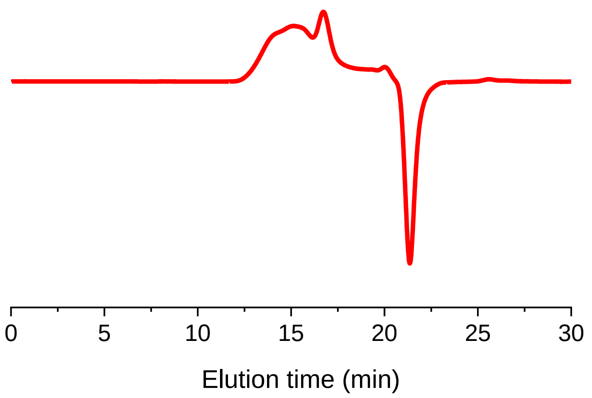

Supplementary Fig. 10 GPC chromatogram of DTPU in DMF/LiBr (2 g L^{-1}).

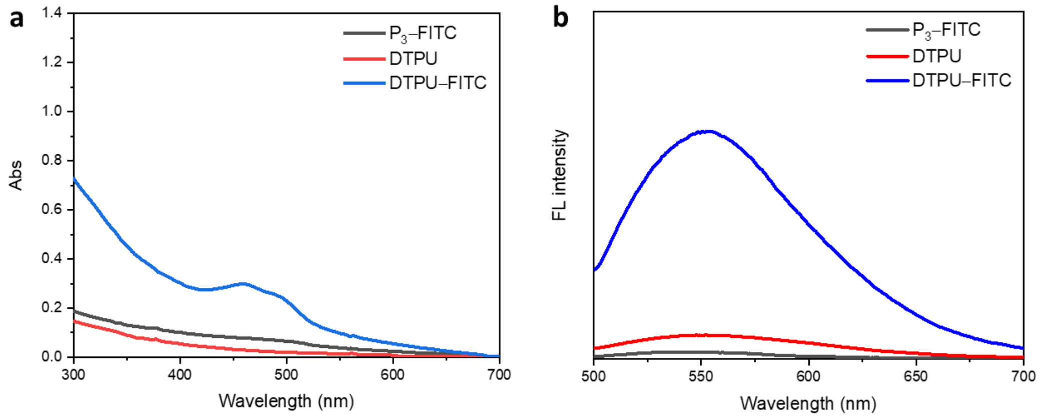

Supplementary Fig. 11 (**a**) UV-vis spectra and (**b**) fluorescence spectra (λ_{ex} = 493 nm) of PU and DTPU assemblies after labelling with FITC.

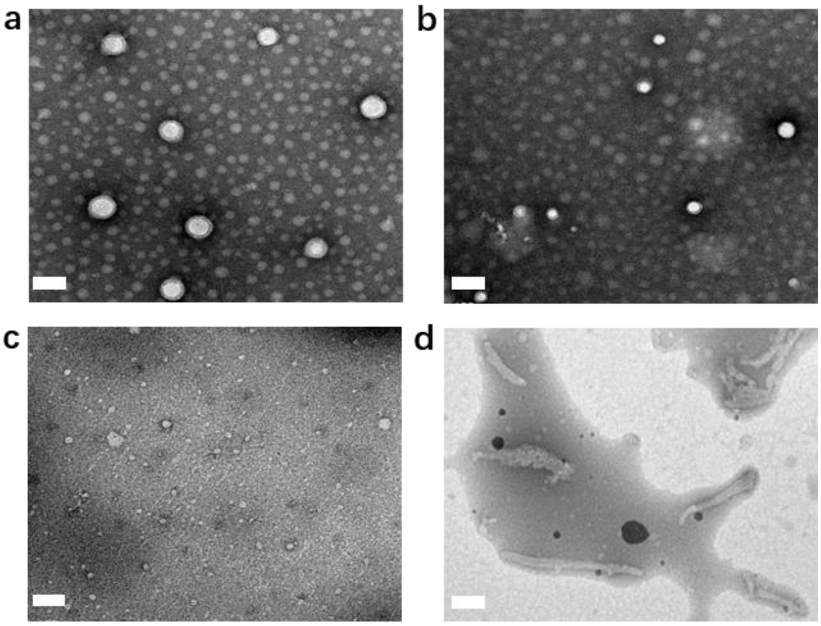

Supplementary Fig. 12 TEM images of PU assemblies: (**a**) P_1; (**b**) P_2; (**c**) P_3 and (**d**) P_4. The scale bars are 100 nm. Experiments were repeated three times independently with similar results.

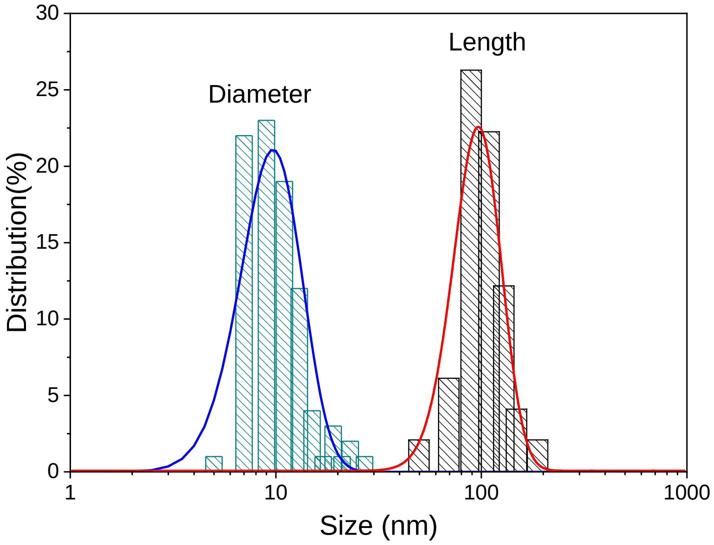

Supplementary Fig. 13 Size (diameter and length) distribution of P_4 assembly obtained from TEM images. The solid line shows Gaussian distribution.

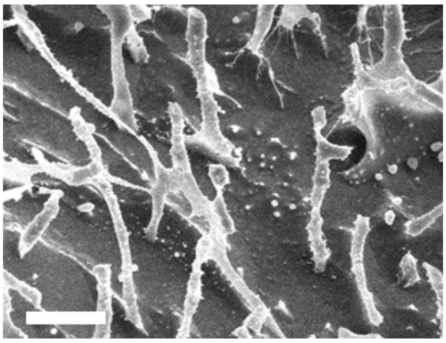

Supplementary Fig. 14 cryo-SEM image of P_4 assemblies. The scale bar is 100 nm. Experiments were repeated three times independently with similar results.

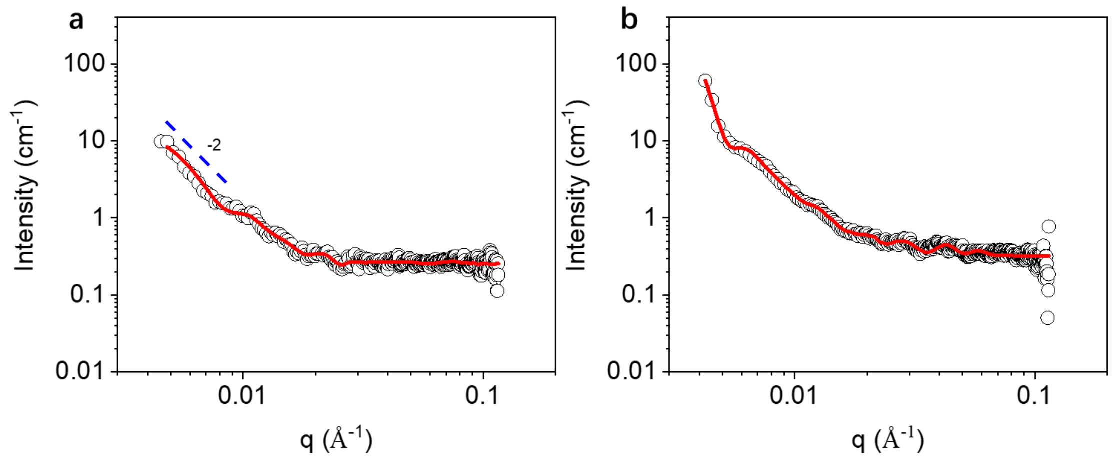

Supplementary Fig. 15 SAXS results of (**a**) P_2 and (**b**) P_4 assemblies in water. The experimental data were plotted in black, and the calculated curves were plotted in red.

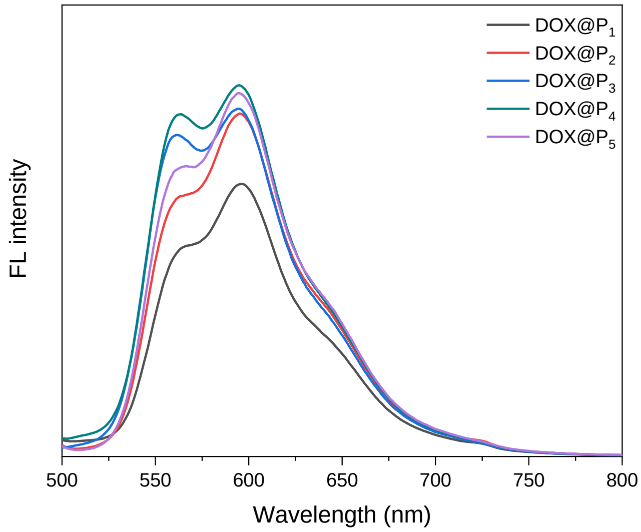

Supplementary Fig. 16 Fluorescence emission spectra of DOX-encapsulated PU assemblies (DOX@PUs).

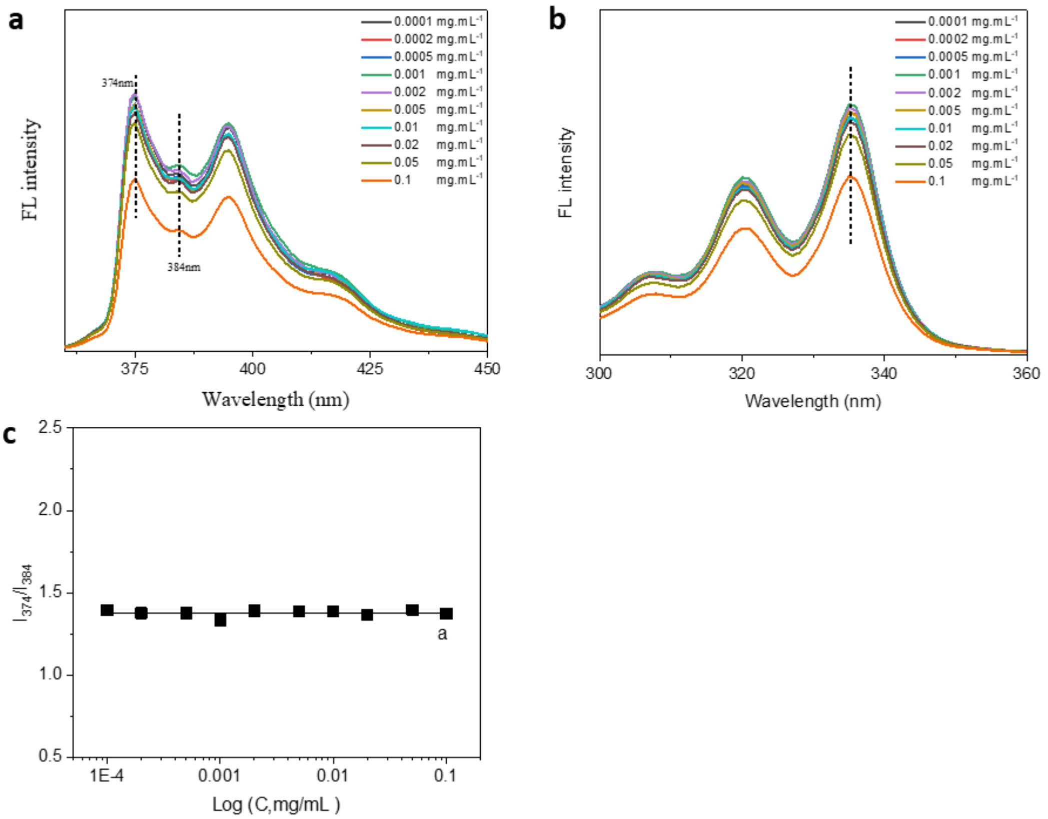

Supplementary Fig. 17 Pyrene fluorescence spectra and I_{374}/I_{384} values of pyrene in PU assemblies with different concentration: (**a**) emission spectra (λ_{ex} = 334 nm), (**b**) excitation spectra (λ_{em} = 372 nm), (**c**) I_{374} / I_{384} ratios from (**c**) as a function of the concentrations (log C) of PU assemblies.

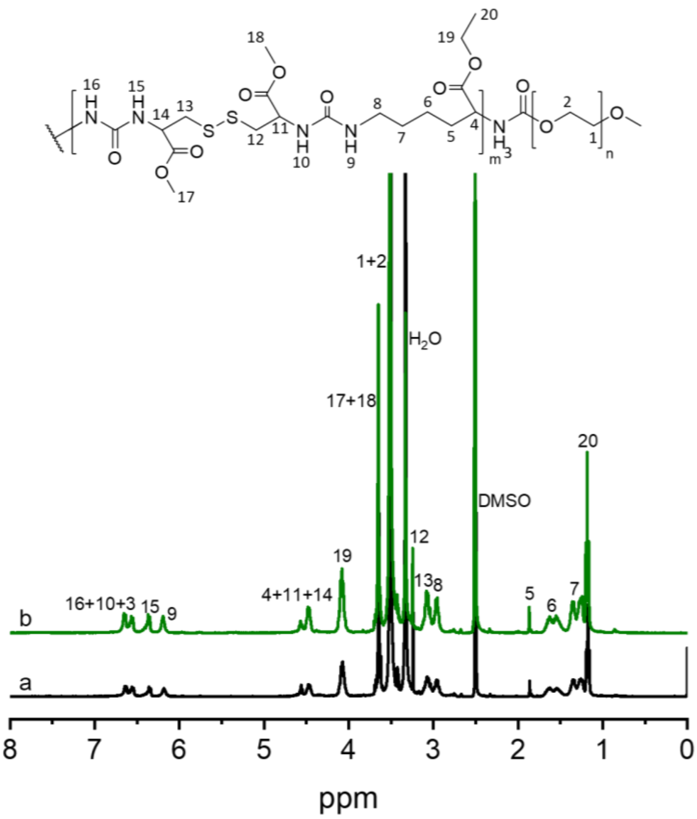

Supplementary Fig. 18 ^{1}H NMR spectra (400 MHz) of P_3 in DMSO-d_6 before (**a**) and after TFA treatment (**b**).

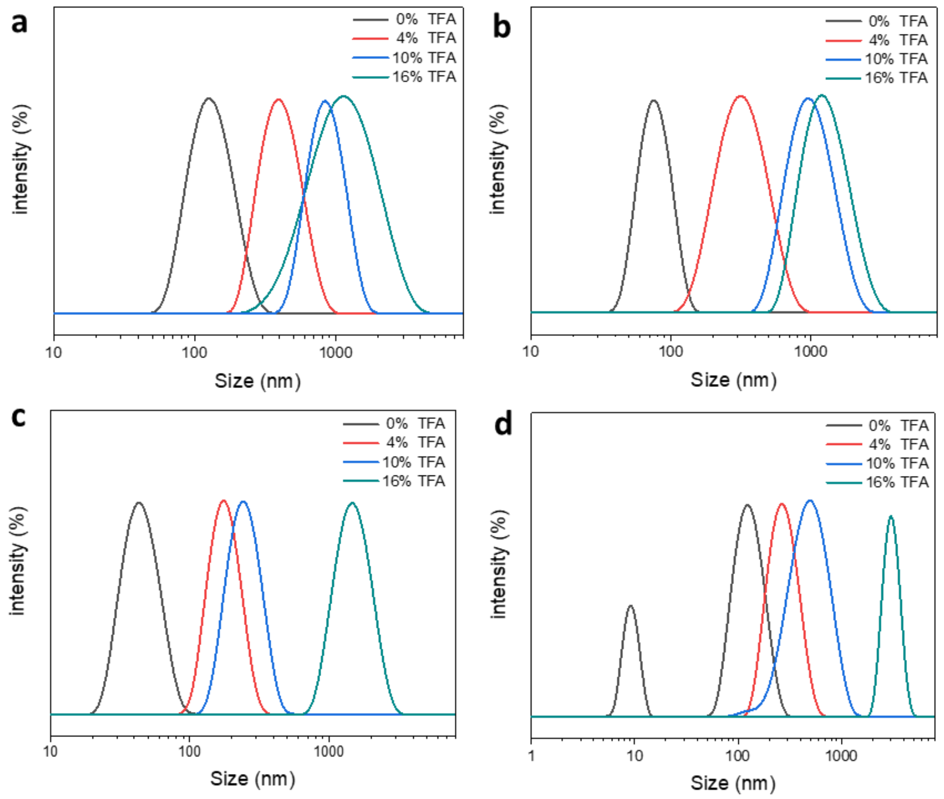

Supplementary Fig. 19 Size distribution curves of PU assemblies in aqueous solutions containing different concentrations of TFA (0, 4, 10, 16 *v*%): **(a)** P_1; **(b)** P_2; **(c)** P_3; **(d)** P_4.

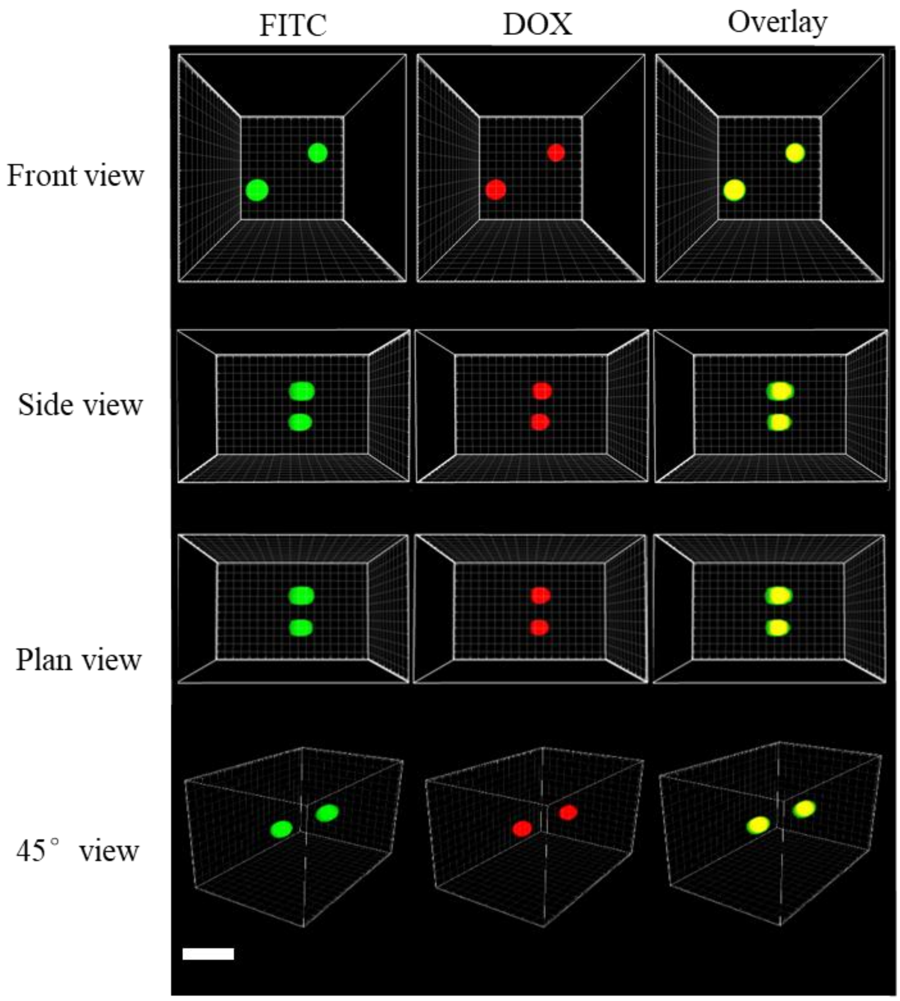

Supplementary Fig. 20 3D confocal micrographs of FITC- and DOX-coloaded PU assemblies from different perspectives. Volume dimension = 24 × 24 × 36 μm. The scale bar is 4 μm.

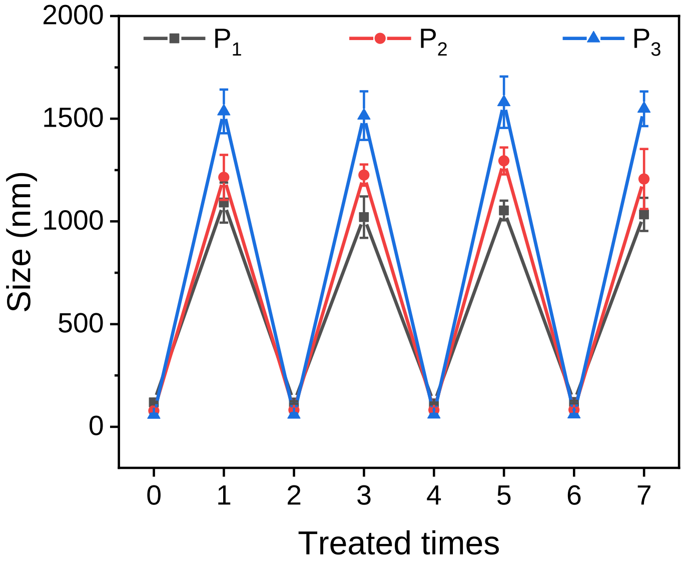

Supplementary Fig. 21 Particle sizes of PU assemblies before and after TFA treatments determined by DLS. TFA (16 *v*%) was added and removed repeatedly before measurements. Data are presented as the mean ± SD ($n = 3$ independent measurements).

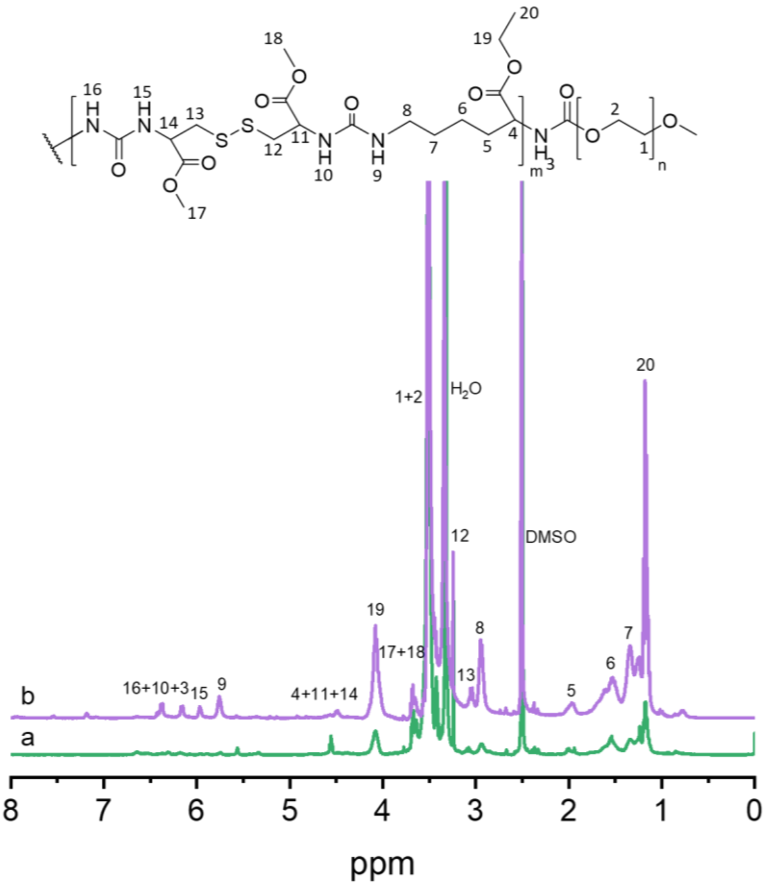

Supplementary Fig. 22 ^{1}H NMR spectra (400 MHz) of DL-PUs in DMSO-d_6: (**a**) DL-P_1; (**b**) DL-P_4.

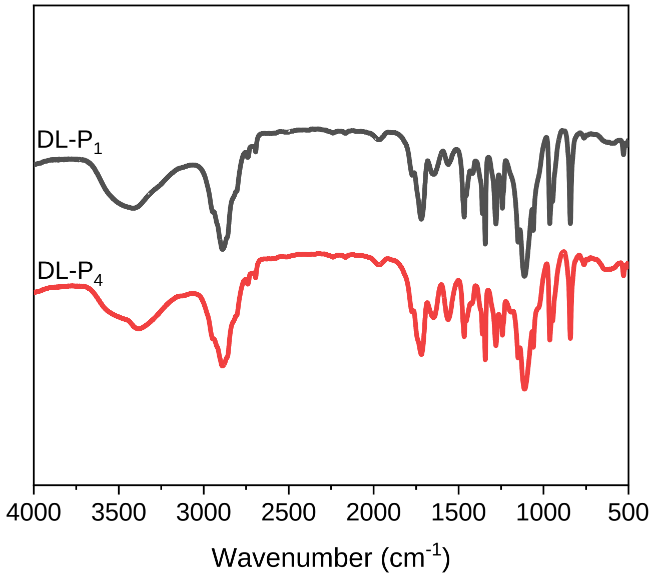

Supplementary Fig. 23 FTIR spectra of DL-PUs (500-4000 cm^{-1}).

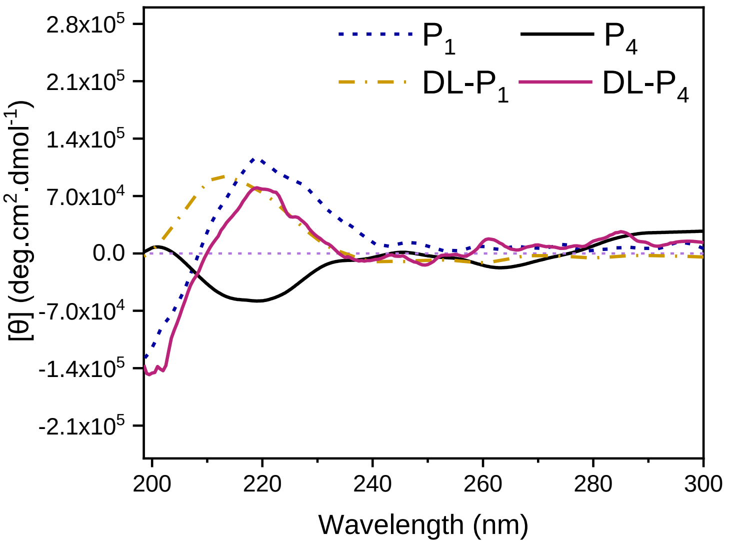

Supplementary Fig. 24 CD spectra of PU and DL-PU assemblies.

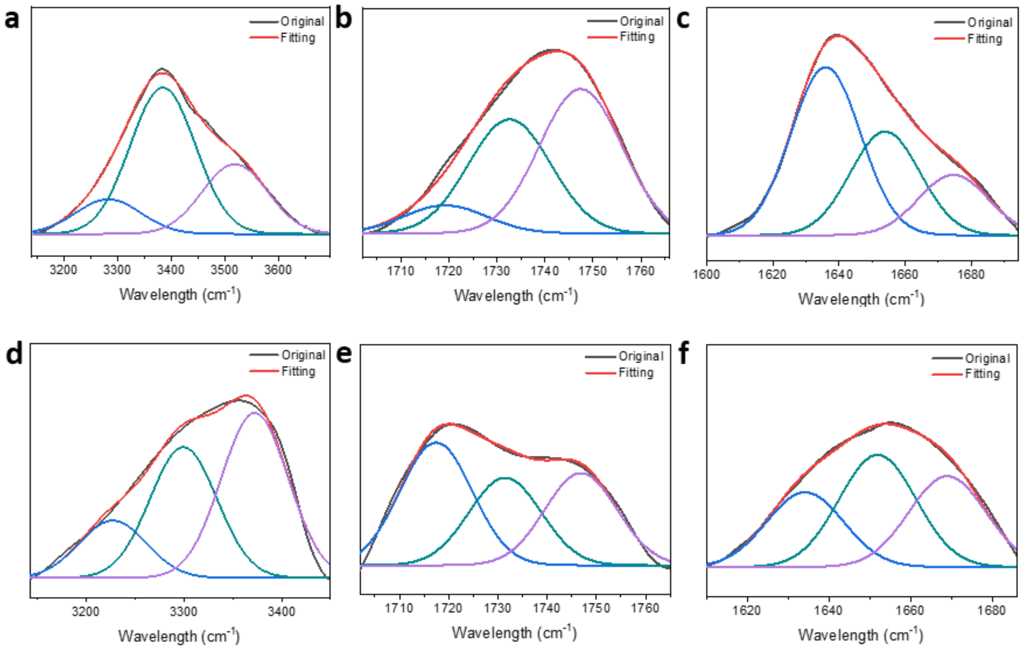

Supplementary Fig. 25 FTIR spectra of PUs fitted with gaussian function. NH stretching vibration (3600-3100 cm^{-1}) (**a**, **d**), C=O $_{ester}$ stretching vibration (1770-1690 cm^{-1}) (**b**, **e**) and C=O $_{urea}$ stretching vibration (1690-1600 cm^{-1}) (**c**, **f**) in the FITR spectra of P_3 (**a-c**) and P_4 (**d-f**).

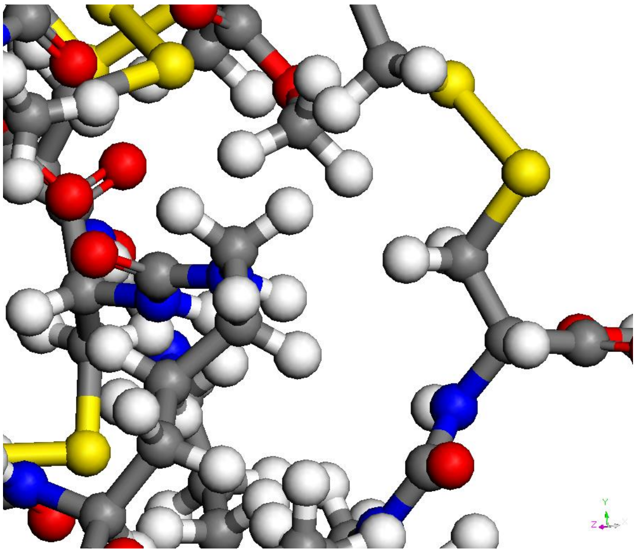

Supplementary Fig. 26 Structure of P_2 obtained from MD simulations.

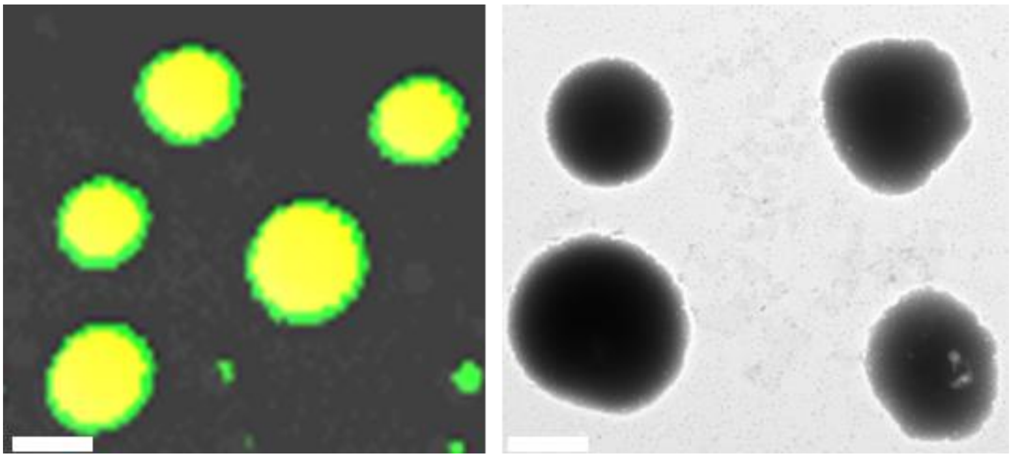

Supplementary Fig. 27 CLSM (left) and TEM (right) images of P_4 assemblies treated with TFA (16 *v*%). The left and right scale bars are 2 μm and 1 μm, respectively. Experiments were repeated three times independently with similar results.

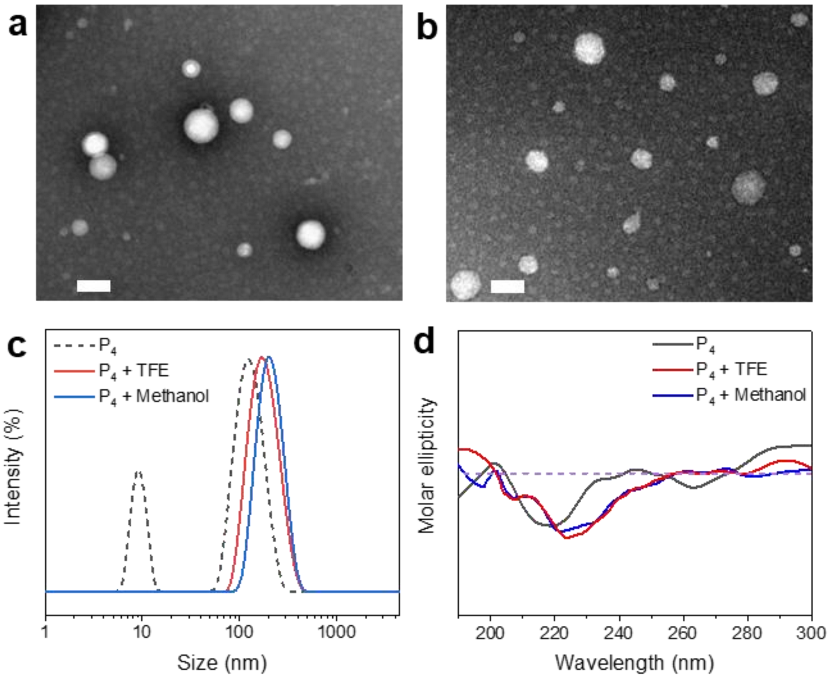

Supplementary Fig. 28 (**a**, **b**) TEM images of P_4 assemblies treated with methanol (**a**) and TFE (**b**). The scale bars are 100 nm. (**c**, **d**) Size distribution profiles (**c**) and CD spectra (**d**) of P_4 assemblies before and after treatments with methanol and TFE.

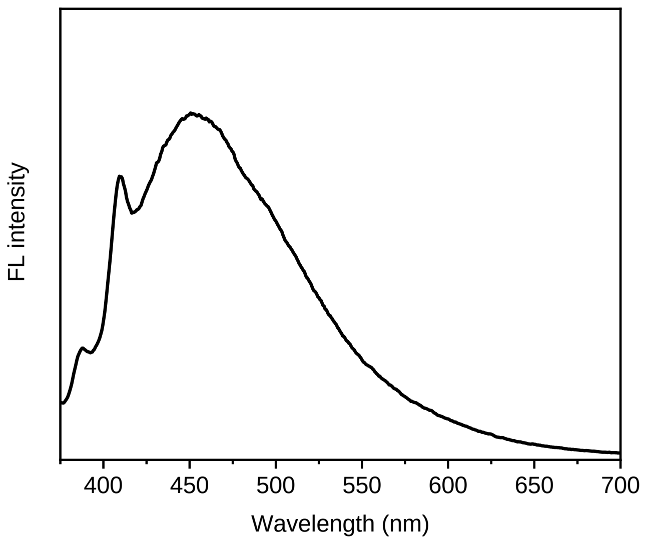

Supplementary Fig. 29 Fluorescence spectrum of P_1 solution in DMF (0.5 mg mL^{-1}) (λ_{ex} = 365 nm).

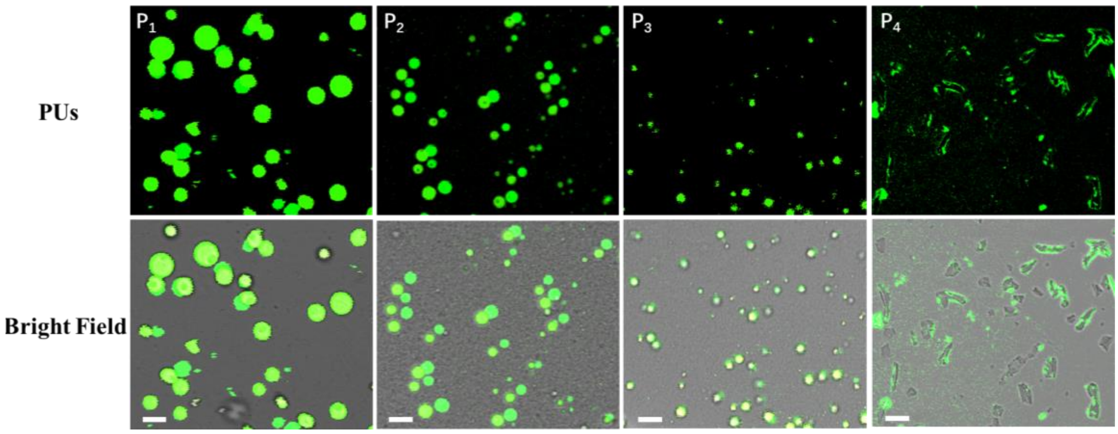

Supplementary Fig. 30 CLSM images of PU assemblies (intrinsic fluorescence: green). The scale bars are 100 nm. Experiments were repeated three times independently with similar results.

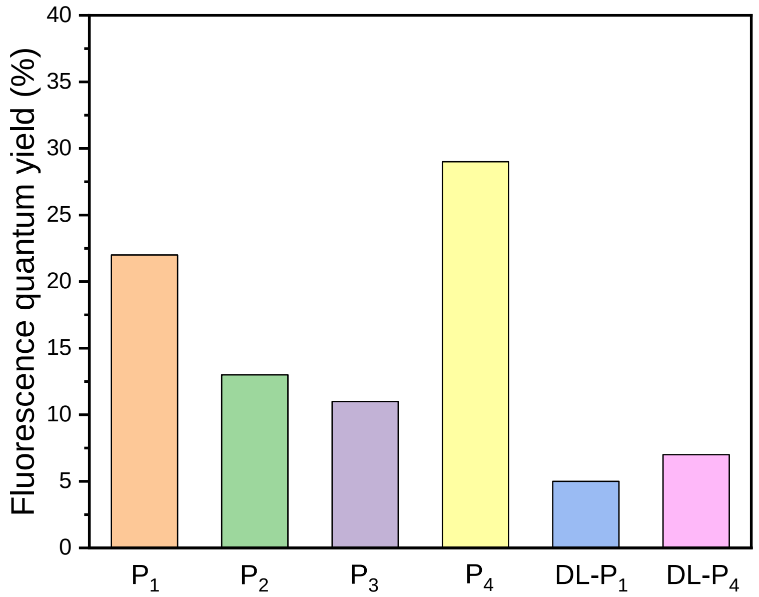

Supplementary Fig. 31 Fluorescence quantum yields of PUs and DL-PUs dissolved in DMF (10 μg mL^{-1}).

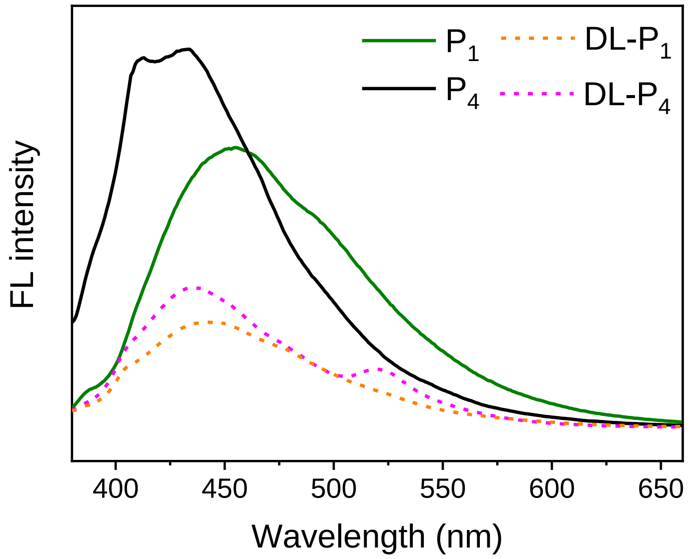

Supplementary Fig. 32 Fluorescence spectra of DL-PU assembles (1 mg mL^{-1}).

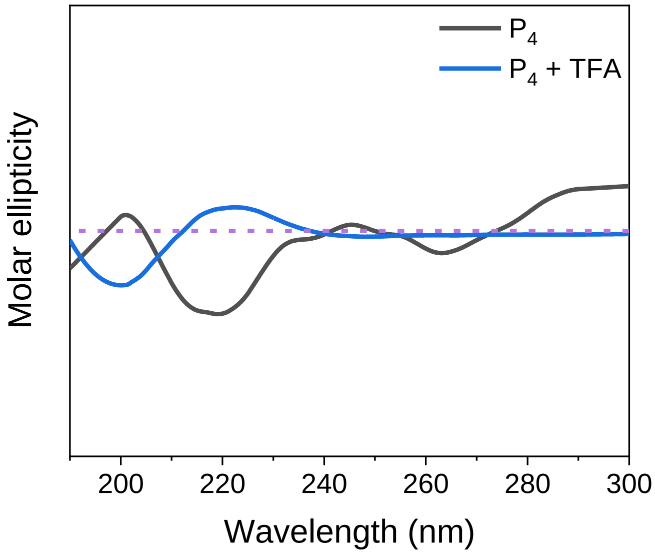

Supplementary Fig. 33 CD spectra of P_4 assemblies before and after TFA treatment.

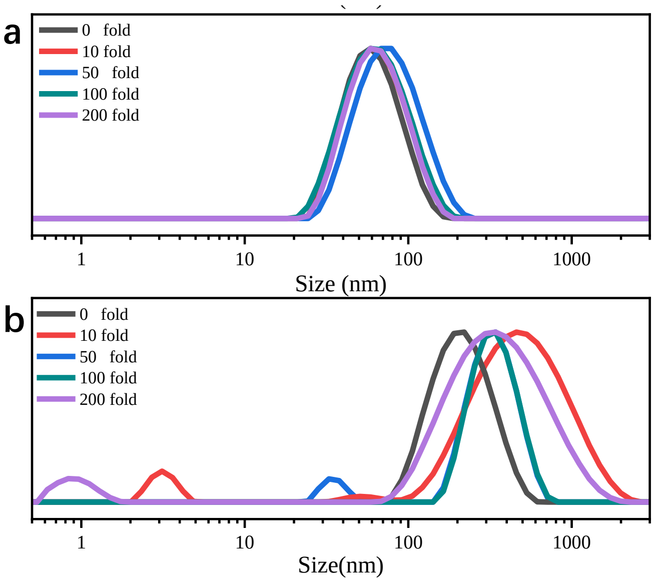

Supplementary Fig. 34 Size distributions of P_3 assemblies (**a**) and mPEG-PCL assemblies (**b**) before (**a**) and after dilution with water for different times. The arabic numbers in the figures indicate dilution factors.

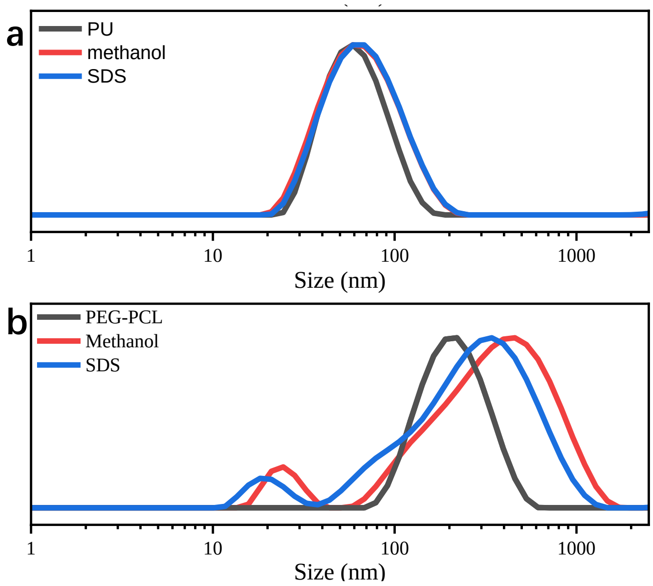

Supplementary Fig. 35 Size distributions of P_3 assemblies (**a**) and mPEG-PCL assemblies (**b**) before and after treatments with methanol or SDS.

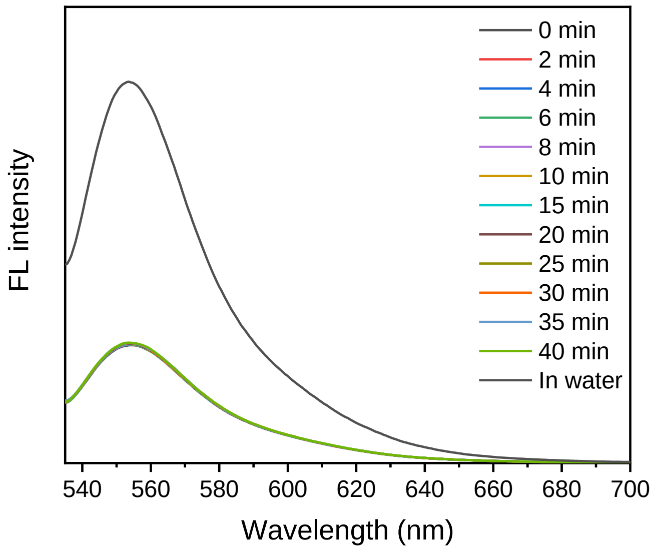

Supplementary Fig. 36 Fluorescence spectra (λ_{ex} = 526 nm) of free R6G dissolved in water and those of R6G-loaded P_3 assemblies incubated for different times in the absence of GSH.

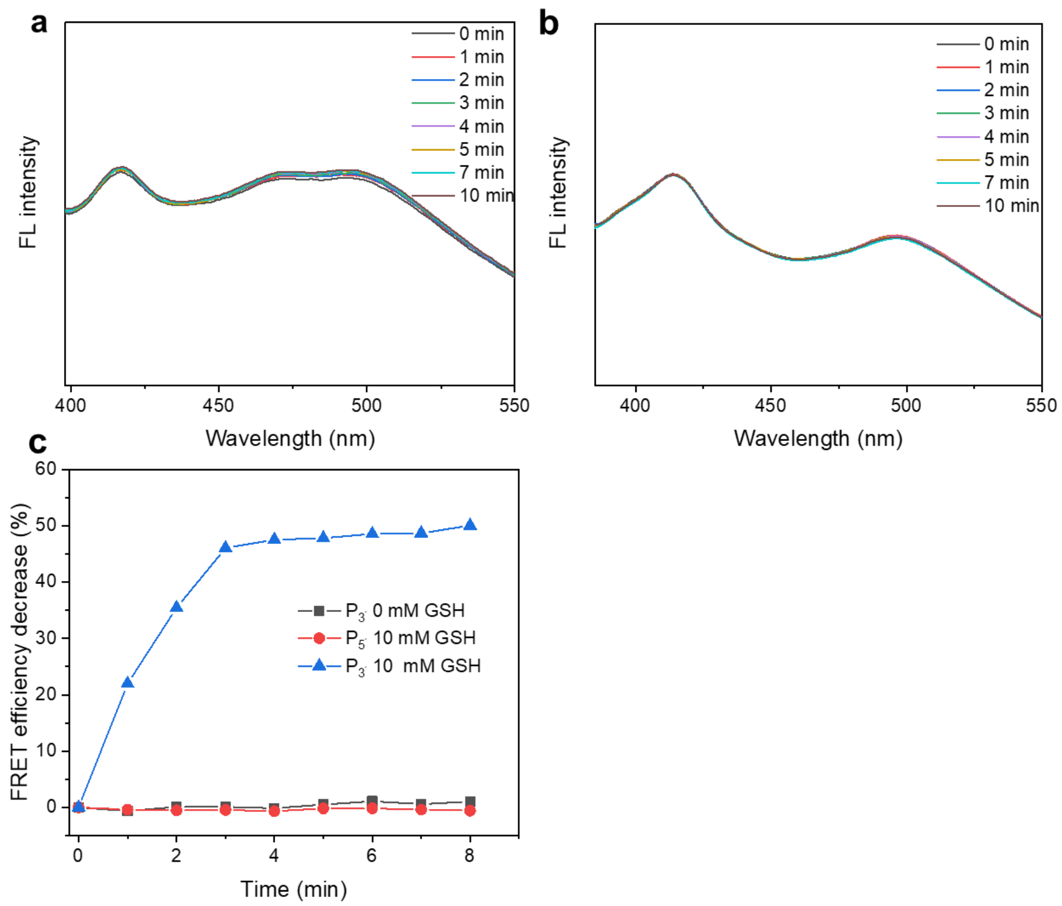

Supplementary Fig. 37 Fluorescence spectra of QD-loaded P_3 assemblies incubated with 0 mM of GSH (**a**) and those of P_5 assemblies incubated with10 mM of GSH (**b**) for different times. (**c**) Normalized decrease in FRET efficiency (%) of QD-loaded PU assemblies in the media with or without 10 mM of GSH.

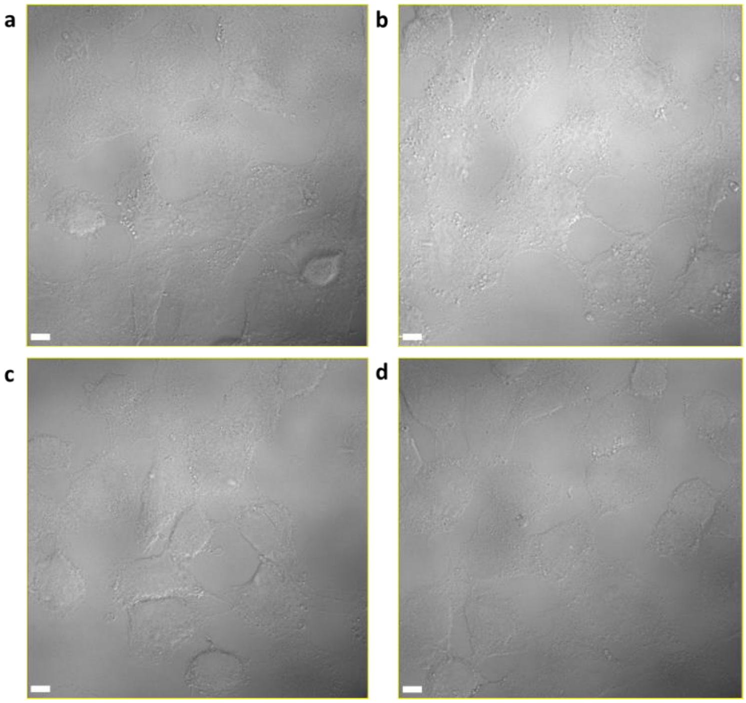

Supplementary Fig. 38 CLSM images (bright field) of MCF-7 tumor cells incubated with PU assemblies for 2 h: (**a**) P_1; (**b**) P_2; (**c**) P_3; (**d**) P_4. The scale bars are 5 μm. Experiments were repeated three times independently with similar results.

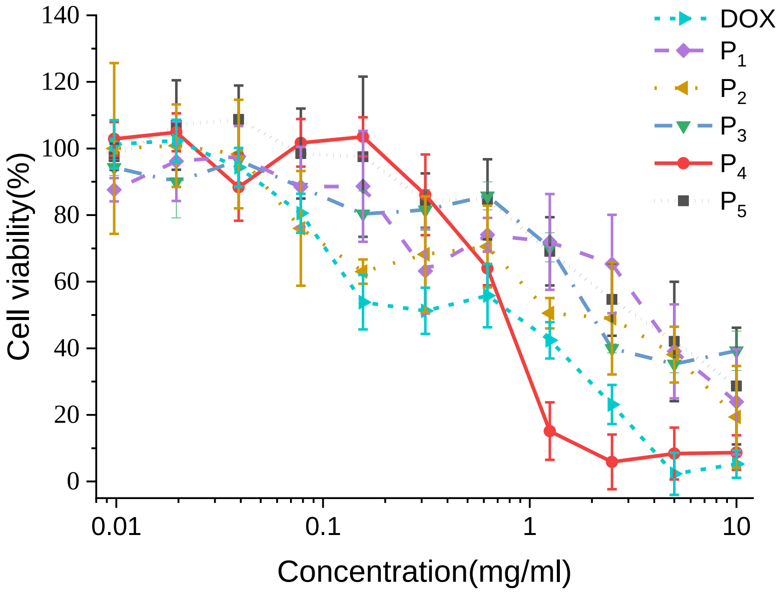

Supplementary Fig. 39 Cell viability of MCF-7 cells incubated with DOX-loaded PU assemblies for 48 h with different concentrations of DOX, setting free DOX as a control. Data are presented as mean ± SD ($n = 3$ independent experiments).

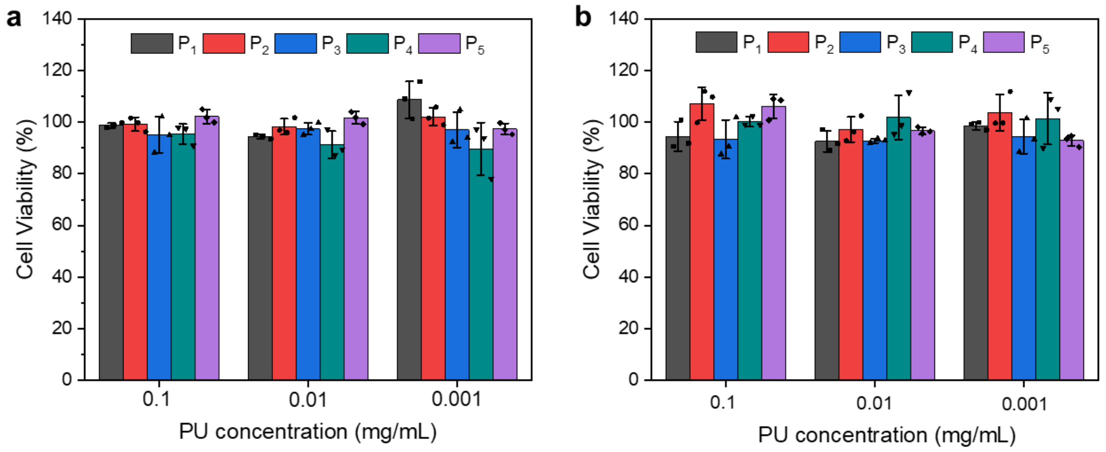

Supplementary Fig. 40 Cell viability of L929 mouse fibroblasts after incubation with PUs at different concentrations for 24 h (**a**) and 72 h (**b**). Data are presented as mean ± SD ($n = 3$ independent experiments).

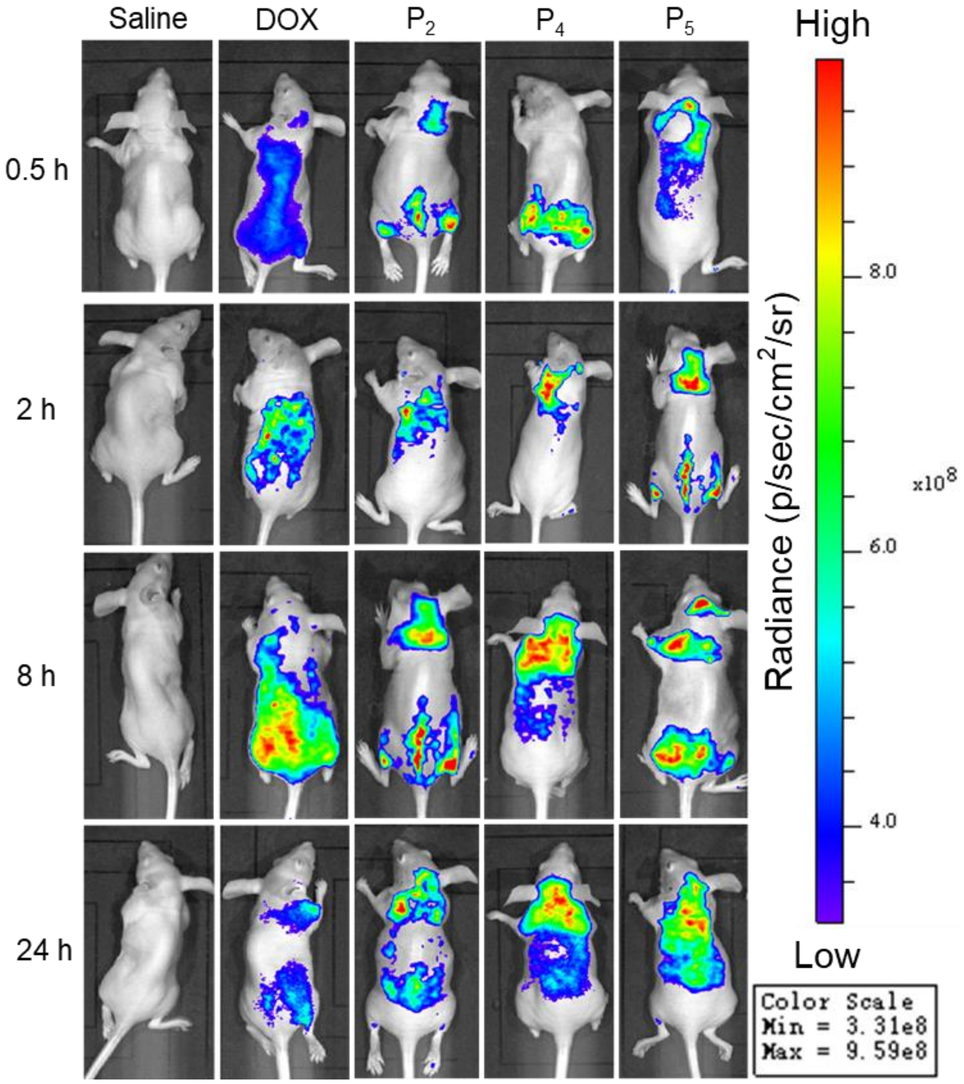

Supplementary Fig. 41 In vivo imaging of MCF-7 tumor-bearing KM mice at different times after intravenous injection of DOX@P_2, DOX@P_4 and DOX@P_5. Mice receiving saline were set as control. λ_{ex} = 480 nm, λ_{em} = 600 nm.

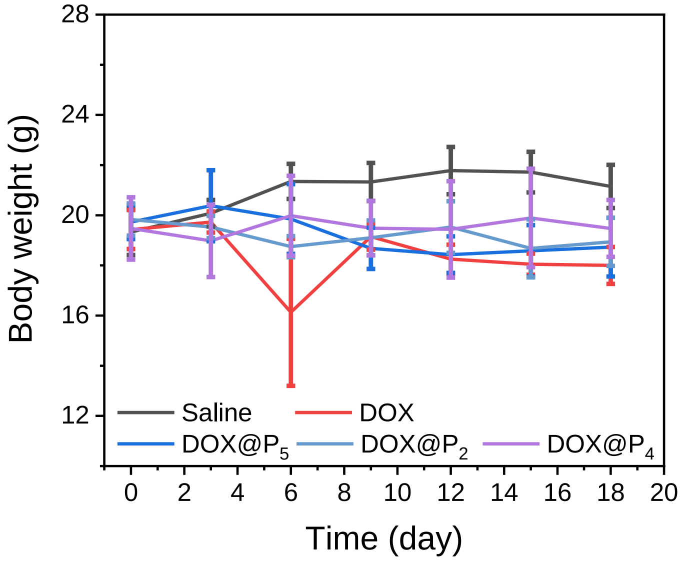

Supplementary Fig. 42 Body weight changes of MCF-7 tumor-bearing nude mice after treatment with saline, DOX, and DOX-loaded PU assemblies (DOX@P_2, DOX@P_4 and DOX@P_5) at a DOX dose of 5 mg kg^{-1}. Data were presented as the mean ± SD ($n = 6$ independent animals per group).

Supplementary Tables

Supplementary Table 1. Calculated values based on the integrated areas of the identified peaks in the ^{1}H-NMR spectra of PUs.

Chemical shifts (ppm)	Assignments	Integration values						
		P_1	P_2	P_3	P_4	P_5	DL-P_1	DL-P_4
4.08	COO-C$\boldsymbol{H}_2$-CH_3 (LDI residues)	22.5	32.1	42.6	82.4	62.7	20.3	80.9
3.66	COO-C$\boldsymbol{H}_3$ (Cys OMe residues)	30.4	45.8	60.2	120.1	—	27.5	117.3
3.50	mPEG	452	452	452	452	452	452	452

Supplementary Table 2. Characteristics of the free and H-bonded carbonyl bands in the infrared spectra of the PUs.

Samples		Free C=O (ester)	H-bonded C=O (ester)	Free C=O (urea)	H-bond $_{or}$ C=O (urea)	H-bond $_{disor}$ C=O (urea)
P_3	Percentages	91%	9%	17%	51%	32%
	Wavenumber	1744 cm^{-1}	1719 cm^{-1}	1675 cm^{-1}	1636 cm^{-1}	1654 cm^{-1}
P_4	Percentages	59%	41%	0	27%	73%
	Wavenumber	1744 cm^{-1}	1718 cm^{-1}	0	1635 cm^{-1}	1668 cm^{-1}

Or and disor represent the order and disorder H-bond, respectively. Percentage represents the content of the H-bond.

Supplementary Table 3. The half inhibitory concentration (IC_{50}) of DOX-loaded polymeric self-assemblies.

Sample	DOX	P_4	P_3	P_2	P_1	P_5
IC_{50} (μg mL^{-1})	0.9	1.3	1.8	6.4	6.2	6.9

Supplementary references

1. Li, Z. et al. Healable and recyclable elastomers with record-high mechanical robustness, unprecedented crack tolerance, and superhigh elastic restorability. *Adv. Mater.* **33**, e2101498 (2021).
2. Xing, R. et al. Charge-induced secondary structure transformation of amyloid-derived dipeptide assemblies from beta-sheet to alpha-helix. *Angew. Chem. Int. Ed.* **57**, 1537-1542 (2018).
3. Mattia, J., Painter, P. A comparison of hydrogen bonding and order in a polyurethane and poly (urethane− urea) and their blends with poly (ethylene glycol). *Macromolecules* **40**, 1546-1554 (2007).
4. Bonattini, VH. et al. One-step formation of polyurea gel as a multifunctional approach for biological and environmental applications. *Polym. Int.* **69**, 476-484 (2020).
5. Niu, S. et al. A multifunctional silicon-containing hyperbranched epoxy: controlled synthesis, toughening bismaleimide and fluorescent properties. *J. Mater. Chem. C* **4**, 6881-6893 (2016).
6. Du, Y. et al. Facile one-pot synthesis of novel water-soluble fluorescent hyperbranched poly (amino esters). *RSC adv.* **6**, 88030-88037 (2016).

7. Du, Y. et al. Unanticipated strong blue photoluminescence from fully biobased aliphatic hyperbranched polyesters. *ACS Sustainable Chem. Eng.* **5**, 6139-6147 (2017).

8. Zhang, Y. et al. Electrochromic/electrofluorochromic poly (urea-urethane) bearing oligoaniline and tetraphenylethylene groups: Synthesis, characterization, and H_2O_2 visualized determination. *Dyes Pigm.* **194**, 109594 (2021).

9. Praveen, VK. et al. Self-assembled pi-nanotapes as donor scaffolds for selective and thermally gated fluorescence resonance energy transfer (FRET). *J. Am. Chem. Soc.* **128**, 7542-7550 (2006).

10. Yang, R. et al. Crosslinking induced reassembly of multiblock polymers: addressing the dilemma of stability and responsivity. *Adv. Sci* **7**, 1902701 (2020).

11. Tian, Y. et al. Nanotubes, Plates, and Needles: Pathway-Dependent Self-Assembly of Computationally Designed Peptides. *Biomacromolecules* **19**, 4286-4298 (2018).

12. Guinier, A, Fournet, G., Yudowitch, KL. Small-angle scattering of X-rays. **24** (1955).

13. Mizuta, R. et al. Dynamic self-assembly of DNA minor groove-binding ligand DB921 into nanotubes triggered by an alkali halide. *Nanoscale* **10**, 5550-5558 (2018).

14. Ananthapadmanabhan, K, Goddard, E, Turro, N., Kuo, P. Fluorescence probes for critical micelle concentration. *Langmuir* **1**, 352-355 (1985).

15. Wang, Y-S, Liu, L-R, Jiang, Q., Zhang, Q-Q. Self-aggregated nanoparticles of cholesterol-modified chitosan conjugate as a novel carrier of epirubicin. *Eur. Polym. J.* **43**, 43-51 (2007).

16. Wilhelm, M. et al. Poly (styrene-ethylene oxide) block copolymer micelle formation in water: a fluorescence probe study. *Macromolecules* **24**, 1033-1040 (1991).

17. Astafieva, I, Zhong, XF., Eisenberg, A. Critical micellization phenomena in block polyelectrolyte solutions. *Macromolecules* **26**, 7339-7352 (1993).

18. Cornelissen, JJ. et al. β-helical polymers from isocyanopeptides. *Science* **293**, 676-680 (2001).
