# Supplementary material for: Intrinsically fluorescent polyureas toward conformation-assisted metamorphosis, discoloration and intracellular drug delivery
Source: Nat Commun. 2022 Aug 5;13:4551. doi: 10.1038/s41467-022-32053-1 (PMC9355952; doi:10.1038/s41467-022-32053-1)
Supplement: Supplementary file 2 — Reporting Summary [file 41467_2022_32053_MOESM2_ESM.pdf]

nature portfolio

Corresponding author(s): Mingming Ding

Last updated by author(s): Jun 25, 2022

Reporting Summary

Nature Portfolio wishes to improve the reproducibility of the work that we publish. This form provides structure for consistency and transparency in reporting. For further information on Nature Portfolio policies, see our Editorial Policies and the Editorial Policy Checklist.

Statistics

For all statistical analyses, confirm that the following items are present in the figure legend, table legend, main text, or Methods section.

n/a	Confirmed	
☐	☒	The exact sample size (*n*) for each experimental group/condition, given as a discrete number and unit of measurement
☐	☒	A statement on whether measurements were taken from distinct samples or whether the same sample was measured repeatedly
☐	☒	The statistical test(s) used AND whether they are one- or two-sided<br>*Only common tests should be described solely by name; describe more complex techniques in the Methods section.*
☒	☐	A description of all covariates tested
☒	☐	A description of any assumptions or corrections, such as tests of normality and adjustment for multiple comparisons
☐	☒	A full description of the statistical parameters including central tendency (e.g. means) or other basic estimates (e.g. regression coefficient) AND variation (e.g. standard deviation) or associated estimates of uncertainty (e.g. confidence intervals)
☐	☒	For null hypothesis testing, the test statistic (e.g. *F*, *t*, *r*) with confidence intervals, effect sizes, degrees of freedom and *P* value noted<br>*Give P values as exact values whenever suitable.*
☒	☐	For Bayesian analysis, information on the choice of priors and Markov chain Monte Carlo settings
☒	☐	For hierarchical and complex designs, identification of the appropriate level for tests and full reporting of outcomes
☒	☐	Estimates of effect sizes (e.g. Cohen's *d*, Pearson's *r*), indicating how they were calculated

Our web collection on statistics for biologists contains articles on many of the points above.

Software and code

Policy information about availability of computer code

Data collection	The following software were used for data collection:<br>Microsoft Excel (version 2016), F-4600 FL spectrophotometer (Hitachi, Ltd., Japan), J-1500-150 spectrometer (JASCO Corporation, Japan), Xeuss 2.0 system (Xenocs SA, Grenoble, France), Zetasizer Nano ZS instrument (Malvern Instruments Ltd., UK), confocal laser scanning microscope (CLSM, Olympus FV1000, Nikon A1RMP, Japan), microplate reader (DNM-9602, Nanjing Perlove Medical Equipment Co., Ltd., China), IVIS imaging system (Caliper Life Sciences, USA), GraphPad Prism (version 8.0.2), Proton nuclear magnetic resonance spectroscopy (1H NMR, 400 MHz), carbon-13 nuclear magnetic resonance spectroscopy (13C NMR, 400 MHz), AVANCE III HD spectrometer (400 MHz, JEOL), gromacs package (Version 5.1.2), Nicolet iS10 spectrometer (Thermo Electron Corporation, U.S.A), HLC-8320 (TOSOH Corporation, Japan), SEM (JEOL JSM 7100F SEM, JEOL Ltd, Tokyo, Japan), cryo-SEM (PP3010T cryo-SEM Preparation System, Quorum Technologies, UK), TEM (Tecnai G2 F20 S-TWIN electron microscope), UV2600 spectrophotometer (Techcomp, Ltd., China), Origin 8.5.
Data analysis	Chemical structures were drawn by Chemdraw (version 14.0.0.117)<br>The 1H NMR and 13C NMR spectra were analyzed by MestReNova (version 6.1.0-6224)<br>The FTIR spectra were analyzed by OMNIC (version 8.2.0.387)<br>Fluorescence intensity were quantified with Image J software (version 1.50i)<br>Visual molecular dynamics (VMD 1.9.3) was used to analyse MD data within this study.<br>SAXS data were analyzed by SasView software (version 5.0)<br>Vivo imaging data were analyzed by Living Image software (version 4.4)<br>Statistical analysis and graphic representation: GraphPad Prism (version 8.0.2)

For manuscripts utilizing custom algorithms or software that are central to the research but not yet described in published literature, software must be made available to editors and reviewers. We strongly encourage code deposition in a community repository (e.g. GitHub). See the Nature Portfolio guidelines for submitting code & software for further information.

Data

Policy information about availability of data

All manuscripts must include a data availability statement. This statement should provide the following information, where applicable:

- Accession codes, unique identifiers, or web links for publicly available datasets
- A description of any restrictions on data availability
- For clinical datasets or third party data, please ensure that the statement adheres to our policy

The data that support the findings of this study are available within the paper, Supplementary Information files and Source Data file, or are available from the corresponding author upon request. Source data are provided with this paper.

Field-specific reporting

Please select the one below that is the best fit for your research. If you are not sure, read the appropriate sections before making your selection.

- [x] Life sciences
- [ ] Behavioural & social sciences
- [ ] Ecological, evolutionary & environmental sciences

For a reference copy of the document with all sections, see nature.com/documents/nr-reporting-summary-flat.pdf

Life sciences study design

All studies must disclose on these points even when the disclosure is negative.

Sample size	no sample-size calculation was performed, the sample size refers to references of Angew. Chem. Int. Ed. 2012, 51, 11633-11637, Adv. Sci. 2020, 7, 1902701, J. Am. Chem. Soc. 2018, 140, 6604-6610. and Nat. Nanotechnol. 2019, 14, 799-809.
Data exclusions	no date were excluded, each data explains and verifies opinions of this study.
Replication	to verify the reproducibility of the experimental findings, after repeated experiments, all attempts at replication were successful.
Randomization	All experimental samples or mice were allocated randomly to each groups.
Blinding	In this study, mice were employed. All experiments were conducted with blinding and randomization.

Reporting for specific materials, systems and methods

We require information from authors about some types of materials, experimental systems and methods used in many studies. Here, indicate whether each material, system or method listed is relevant to your study. If you are not sure if a list item applies to your research, read the appropriate section before selecting a response.

Materials & experimental systems

n/a	Involved in the study	
x		Antibodies
	x	Eukaryotic cell lines
x		Palaeontology and archaeology
	x	Animals and other organisms
x		Human research participants
x		Clinical data
x		Dual use research of concern

Methods

n/a	Involved in the study	
x		ChIP-seq
x		Flow cytometry
x		MRI-based neuroimaging

Eukaryotic cell lines

Policy information about cell lines

Cell line source(s)	Human breast cancer cell lines (MCF-7 cells) and L929 cells were purchased from West China Medical Center of Sichuan University.
Authentication	STR was used to identify the cells. the detection was carried out on an ABI 3730xl. the peak diagram was read and export data by the machine, and then the data is compared with the database from ATCC, DSMZ, JCRB, KCLB, ECACC.
Mycoplasma contamination	the cell lines were not tested for mycoplasma contamination.
Commonly misidentified lines (See ICLAC register)	we have not used any commonly misidentified cell lines.

Animals and other organisms

Policy information about studies involving animals; ARRIVE guidelines recommended for reporting animal research

Laboratory animals	for laboratory animals, five to six-week-old female BALB/c nude mice were purchased from Vital River Company in Beijing.Mice were housed in groups of six mice per independently ventilated cage for a 12-hour light-dark cycle ((9:00~21:00) light, (21:00~9:00) dark), with a constant ambient temperature (23 ± 1 °C) and relative humidity (50-60%). Food and water were freely available to all mice.
Wild animals	the study did not involve wild animals.
Field-collected samples	the study did not involve samples collected from the field.
Ethics oversight	all experimental procedures were in accordance with the guidelines for laboratory animals established by the Laboratory Animal Center of Sichuan University.

Note that full information on the approval of the study protocol must also be provided in the manuscript.
